# Photoreceptor Outer Segment-like Structures in Long-Term 3D Retinas from Human Pluripotent Stem Cells

**DOI:** 10.1038/s41598-017-00774-9

**Published:** 2017-04-10

**Authors:** Karl J. Wahlin, Julien A. Maruotti, Srinivasa R. Sripathi, John Ball, Juan M. Angueyra, Catherine Kim, Rhonda Grebe, Wei Li, Bryan W. Jones, Donald J. Zack

**Affiliations:** 1 Wilmer Eye Institute, The Johns Hopkins Wilmer Eye Institute 600 N. Wolfe Street, Baltimore, MD 21287 USA; 2 Shiley Eye Institute, University of California San Diego, La Jolla, California USA; 3 Retinal Neurophysiology Section, National Eye Institute, Bethesda, MD USA; 4 Moran Eye Center, University of Utah, Salt Lake City, Utah USA; 5 Department of Molecular Biology and Genetics, Neuroscience, and Institute of Genetic Medicine, Johns Hopkins University School of Medicine, Baltimore, Maryland USA; 6 Is PhenoCell, Evry, France

## Abstract

The retinal degenerative diseases, which together constitute a leading cause of hereditary blindness worldwide, are largely untreatable. Development of reliable methods to culture complex retinal tissues from human pluripotent stem cells (hPSCs) could offer a means to study human retinal development, provide a platform to investigate the mechanisms of retinal degeneration and screen for neuroprotective compounds, and provide the basis for cell-based therapeutic strategies. In this study, we describe an *in vitro* method by which hPSCs can be differentiated into 3D retinas with at least some important features reminiscent of a mature retina, including exuberant outgrowth of outer segment-like structures and synaptic ribbons, photoreceptor neurotransmitter expression, and membrane conductances and synaptic vesicle release properties consistent with possible photoreceptor synaptic function. The advanced outer segment-like structures reported here support the notion that 3D retina cups could serve as a model for studying mature photoreceptor development and allow for more robust modeling of retinal degenerative disease *in vitro*.

## Introduction

Retinal degenerative diseases, such as retinitis pigmentosa (RP) and Leber congenital amaurosis (LCA), are genetic conditions that cause dysfunction and death of photoreceptor (PR) cells, leading to vision loss and often blindness. There has been major progress in defining the over 100 genes that when mutated can cause retinal degeneration (https://sph.uth.edu/retnet/), and there have been important advances in the development of novel therapeutic strategies, such as gene therapy-based treatment approaches for diseases such as LCA^1^. However, despite these impressive advances, many gaps remain in our understanding of the molecular mechanisms of PR loss, and retinal degenerations remain essentially untreatable. Improvements in the directed differentiation of pluripotent stem cell (PSC) derived retinal cells have shown that PSCs can differentiate into retinal pigment epithelium (RPE) and retinal neurons, including PR cells^2–15^, and can give rise to 3D ‘mini-retinas’, organized into self-forming multi-layered retinal tissues^6, 14–21^. During embryogenesis, the eyefield first appears as an optic vesicle (OV) that evaginates outward from the diencephalon. The distal tip then invaginates to form a double layered optic cup (OC) with the outer layer becoming RPE and inner layer becoming neural retina (NR). Rather than optic cups, our stem cell derived 3D retinas are single layered sheets of neuroepithelium much like optic vesicles. These don’t invaginate and lack an optic stalk as well as adjacent RPE (RPE often forms but not directly adjacent to the developing PRs). As these vesicles mature we simply refer to them as “retina cups” (RCs) or “mini-retinas”. These 3D mini-retinas offer exciting new opportunities to study the mechanisms of retinal degeneration, and also provide new models for drug discovery and cell-based therapeutics.

The use of PSC-derived retinal cells to explore the etiology of retinal degeneration is supported by several recent studies^18, 19, 22–25^. One limitation, however, is that such models generally show considerable variability in their shapes, sizes and composition and it has yet to be demonstrated that such cells can be used to reliably model retinal diseases in which the onset of degeneration might be slow or when the phenotype is subtle. More importantly, well-developed photoreceptor outer segments (POS), an essential component of phototransduction, have yet to be demonstrated *in vitro*. For hPSC-derived 3D RC-derived “disease-in-a-dish” models of retinal degeneration to be useful and generally accepted by the research community, key goals have been to decrease variability among RC preparations and to achieve greater retinal maturity in culture.

In this work, we have modified the forced aggregate protocol for generating hPSC-derived 3D retinas and demonstrate that this protocol can support layer specific lamination and that the resulting mini-retinas give rise to PRs, including rods and cones, with robust outer segment-like structures^26^. Our method leads to floating 3D optic vesicle-like structures (OVs) within 12 days and in long-term cultures, leads to advanced PR development including rod and cone outer segments and ribbon synapses, neurotransmitter expression, and membrane conductances and synaptic vesicle release properties consistent with possible photoreceptor synaptic function. Taken together, these advances offer a methodology for studying 3D retina morphology that could offer a means to study late onset retinal degenerative diseases.

## Results

### Stem cell forced aggregates spontaneously give rise to optic vesicles

**Figure 1:**
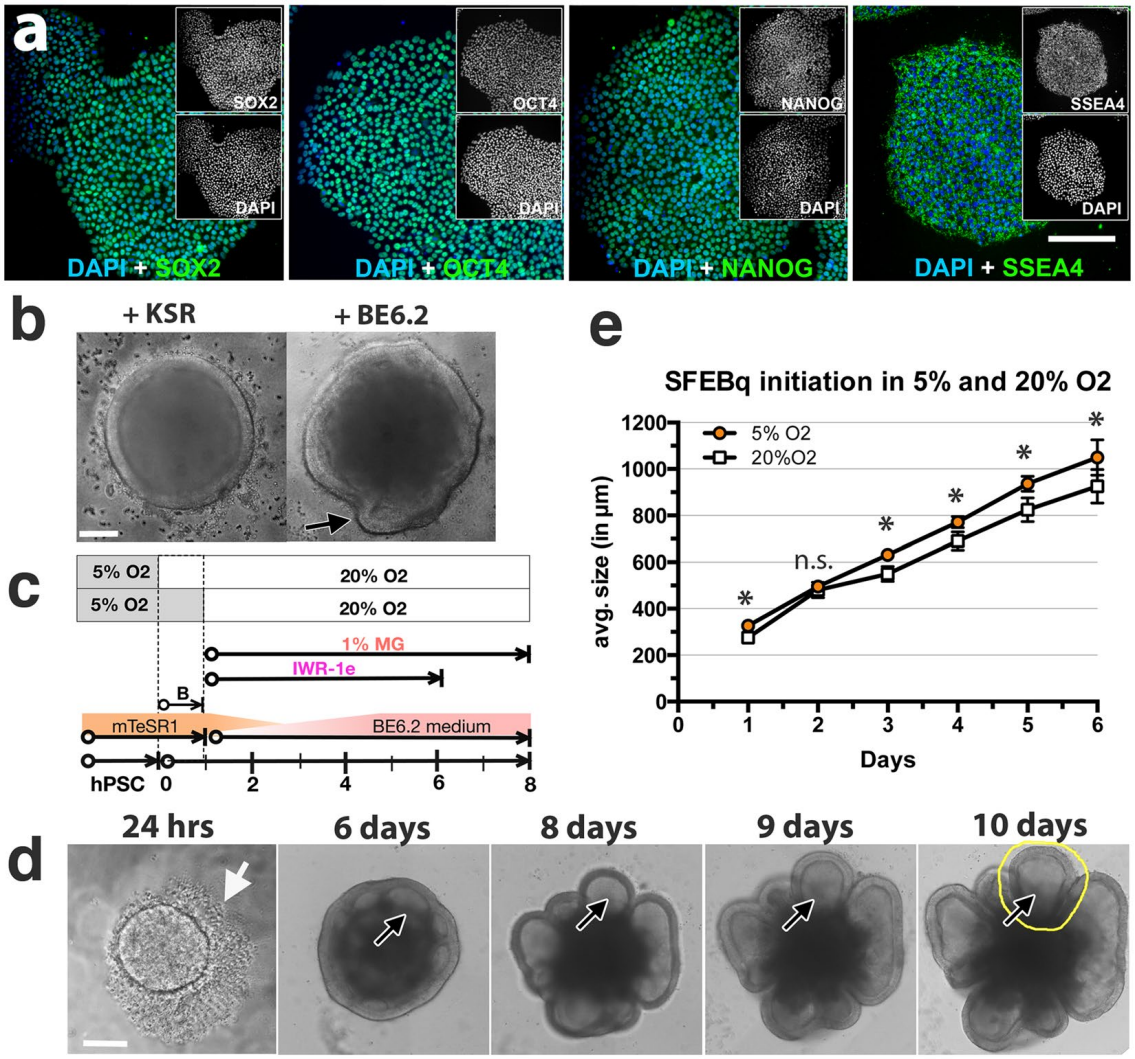
Aggregation of stem cells under hypoxia. (**a**) Pluripotency of IMR90.4 iPSCs confirmed by expression of SOX2, OCT4, NANOG and SSEA4. (**b**) Forced aggregates at day 5 (D5) grown in KSR based neural induction medium (NIM) or BE6.2 medium based NIM in 20% O_2_. Arrow in the BE6.2 sample indicates early vesicle formation. (**c**) Diagram illustrating the approach used to study 1-day recovery and aggregation of dissociated PSCs in 5 or 20% O_2_ followed by 20% O_2_. (**d**) A time-lapse sequence of images taken after 24 hours and from D6-10 illustrating initiation and elaboration of individual vesicles (see dark arrows). In yellow is a typical region selected for manual excision. (**e**) Forced aggregates (n = 15 per time point) in 5% and 20% O_2_ imaged at daily increments, measured along their radial diameter and quantitated. Average diameters plotted as a function of days in culture and statistical significance determined using the student t-test with Holm-Sidak method. *Statistically significant (p < 0.05), n.s. = not significant. Scale bars (**a**) = 200 μm; (**b**,**d**) = 300 μm.

**Figure 2:**
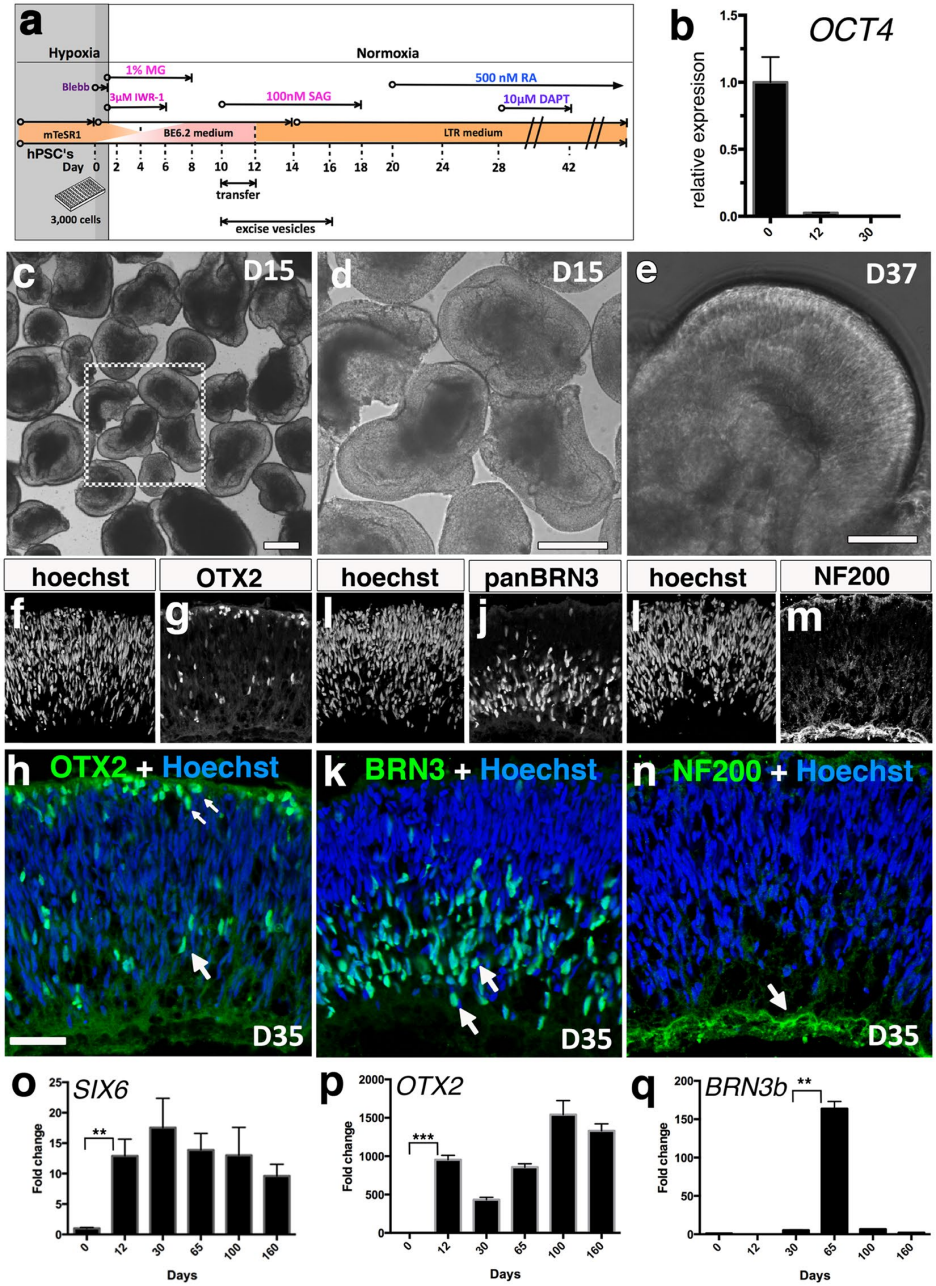
Bulk isolation of optic vesicle-like structures and early characterization in IMR90.4 iPSCs. (**a**) The treatment leading to optic vesicles and retina cups. (**b**) Quantitative PCR analysis of OCT4. (**c**) Cup shaped vesicles at D15 are separated from non-cup shaped vesicles. (**d**) Viewed at higher magnification, is a 3D retinal cup. (**e**) At D37 RC’s maintain their cup-like morphology. (**f**–**h**) Tissue sections from vesicles probed with antibodies against OTX2 at D35 label retinal progenitors and possibly PRs. (**i**–**k**) BRN3+ cells mark RGC’s in the inner retina. (**l**–**n**) NF200 labels the entire retina with enhanced labeling of RGC axon bundles. (**f**,**i**,**l**) Hoechst was used as a nuclear counterstain. (**o**,**p**,**q**) Quantitative PCR analysis of the early retinal markers SIX6, OTX2, and BRN3 was performed on cDNA samples pooled (n = 10) from differentiated IMR90.4 iPSCs to confirm their expression. Data from pooled samples (n = 10 per time point) was calculated using three replicates per time point and error bars represent s.e.m. The difference between the indicated time points is statistically significant by Student’s t-test where ***P < 0.001, **P < 0.01, ns, not significant. PR = photoreceptor; RC = retina cup; RGC = retinal ganglion cell. Scale bars (**a**,**b**) = 500 μm; (**c**) = 200 μm; (**d**) = 100 μm.

**Figure 3:**
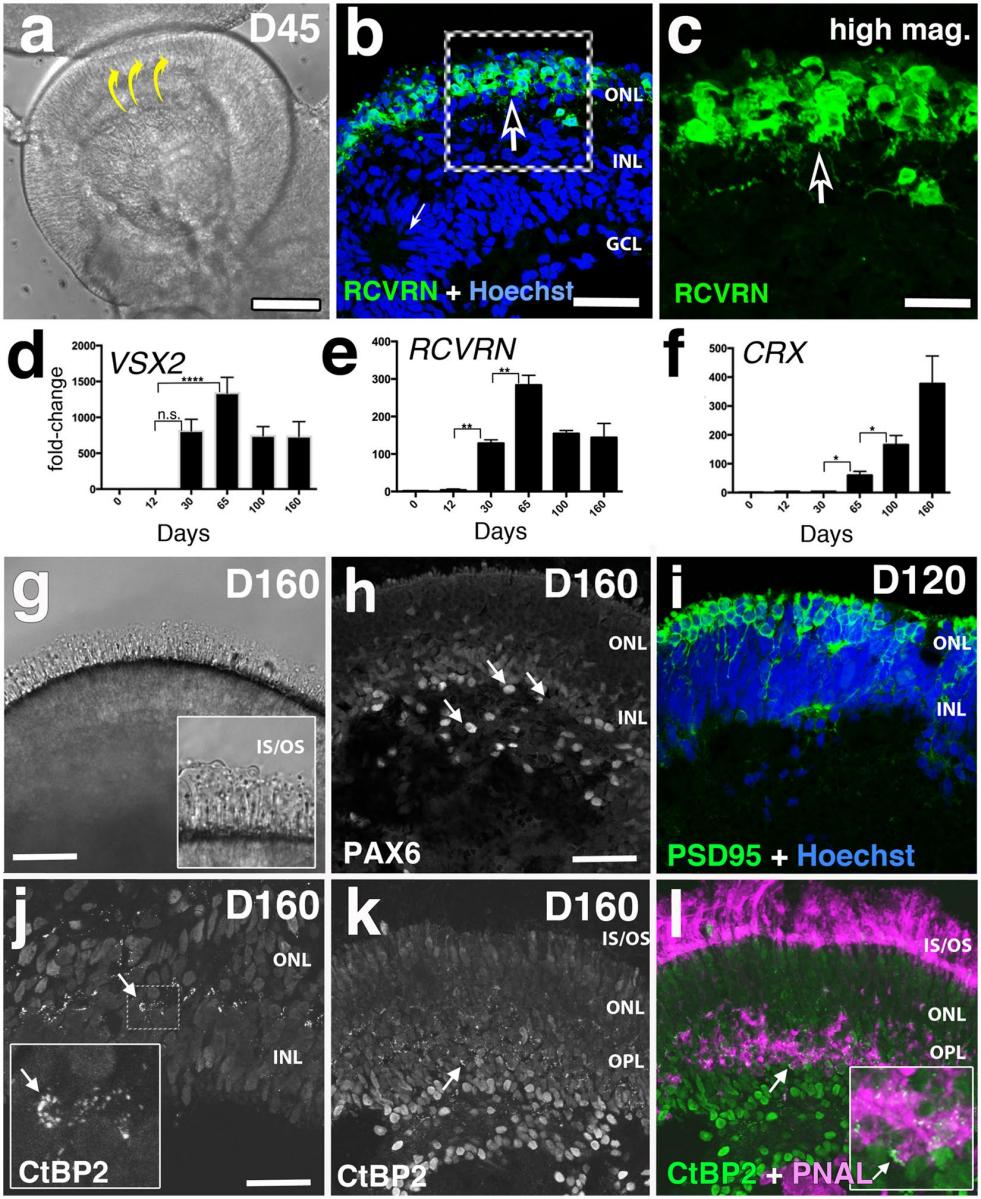
Detection of early photoreceptors after 45 days and markers typical of the neural retina derived from IMR90.4 iPSCs. (**a**) A D45 RC culture with a curved (yellow arrows) pseudostratified appearance. (**b**,**c**) RCVN (+) PRs at D45 line the prospective outer retina. (**d**–**f**) Quantitative PCR analysis of the early retinal markers VSX2, RCVRN, and CRX was performed on cDNA samples pooled (n = 10) from IMR90.4 iPSCs to confirm their relative levels of expression. (**g**) At D160 OS-like structures were observed protruding from the surface of RCs. A higher magnification image of the POSs illustrates the fine radial architecture (inset box). (**h**) PAX6 immunolabeling at D160 labels neurons in the presumptive INL and GCL (arrows). (**i**) PSD95 immunolabeling at D120 in the outer retina where PRs are located. (**j**–**l**) CtBP2 staining in nuclei and synapses in D160 retina is highlighted by arciform structures corresponding to ribbon synapses (**j**, inset panel). (**j**) The position of CtBP2 labeling at synapses occurs at the base of photoreceptor terminals, a position verified by double labeling with PNAL, a marker for PR inner segments and synaptic terminals (**k**,**l**; arrow). An inset box in (**l**) corresponds to the region marked by the arrow in panels (**k** and **l**). Data from pooled samples (n = 10 per time point) was calculated using three replicates per time point and error bars are represented as s.e.m. The difference between the indicated time points is statistically significant by Student’s t-test where ****P < 0.0001, ***P < 0.001, **P < 0.01, *P < 0.05; ns, not significant. INL = inner nuclear layer; OS-like = outer segment-like; PNAL = peanut lectin agglutinin; POS = photoreceptor outer segment; PR = photoreceptor, RC = retina cup; RGC = retinal ganglion cell. Scale bars (**a**) = 300 μm, (**b**) = 50 μm, (**c**) = 25 μm, (**g**) = 40 μm, (**h**,**i**,**k**,**l**) = 60 μm, (**j**) = 50 μm.

**Figure 4:**
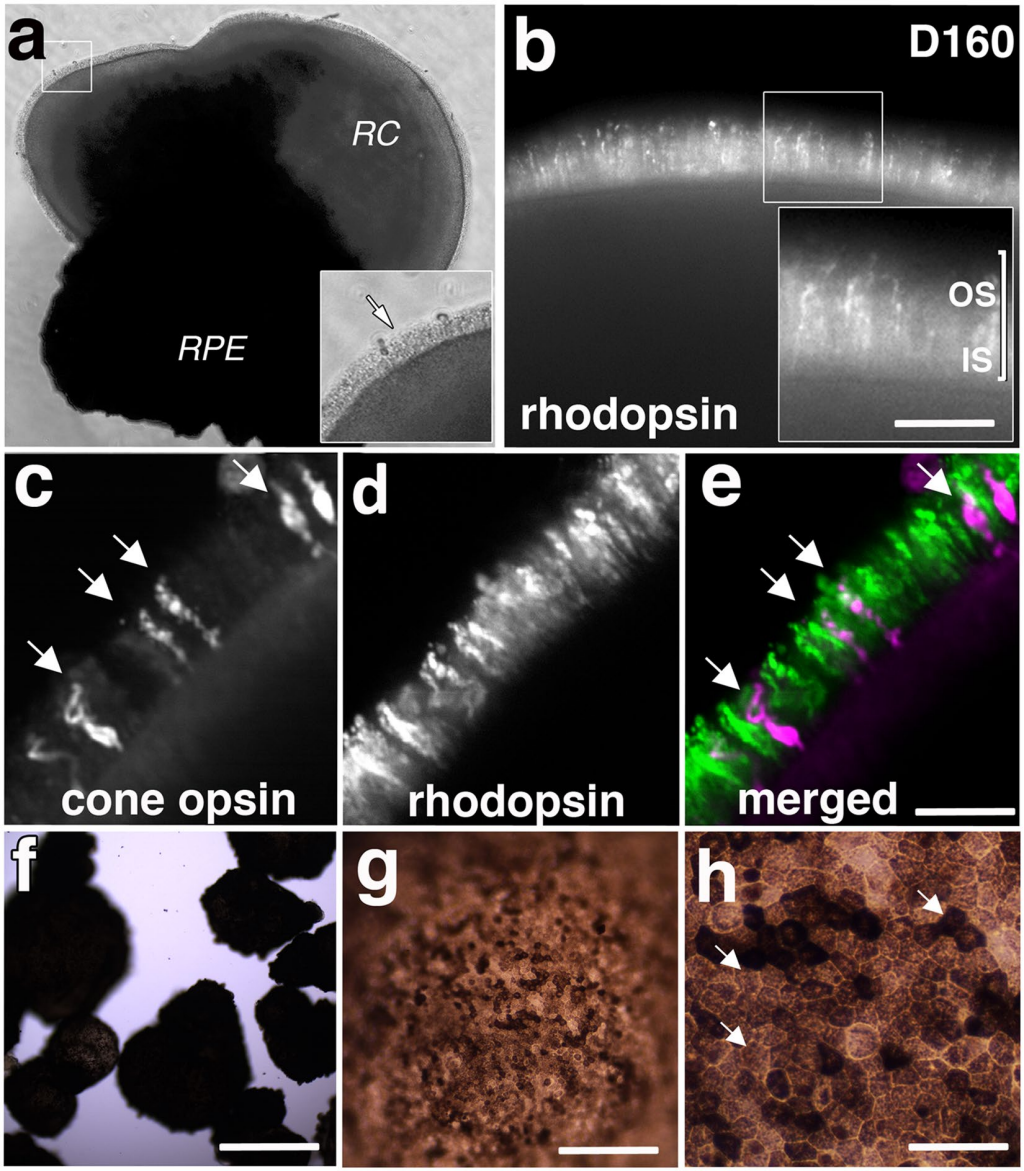
Photoreceptor outer segments express visual pigments in 160 day-old retina cups from IMR90.4 iPSCs. (**a**) A RC with a dense patch of RPE and POS-like structures (arrow). Low (**b**) and high (inset) magnification images of RC whole mounts with broad expression of rhodopsin in cells lining the edge of the RC. Cones (**c**; arrows) and rods (**d**) with discrete non-overlapping expression (**e**) patterns are confirmed by whole mount IHC using antibodies against rhodopsin and R/G cone opsin respectively. (**f**–**h**) Clusters of pigmented RPE grow as spheroid clumps within the culture dish. At high magnification (**h**) these appear as thin sheets of cuboidal shaped cells (arrows). POS = photoreceptor outer segment; RC = retina cup, RPE = retinal pigment epithelium. Scale bars (**b**) = 35 μm, (**e**) = 50 μm, (**f**) = 500 μm, (**g**) = 200 μm, (**h**) = 50 μm.

**Figure 5:**
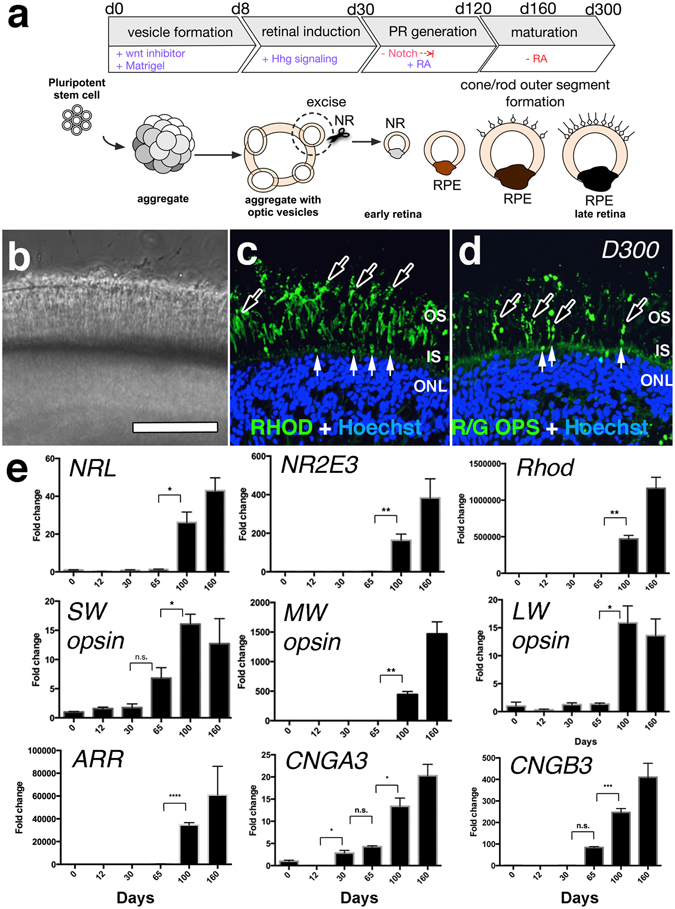
Localization of cone and rod opsins is restricted to outer segments late in Day 300 retina cups derived from IMR90.4 iPSCs. (**a**) Schematic diagram of the important stages during the transition from pluripotent stem cells into 3D retina cups bearing rod and cone outer segments. (**b**) A brightfield image of exuberant POS growth from the surface of RCs. The distal tips of POS are prominently labeled with antibodies against (**c**) rhodopsin and (**d**) red-green opsin. Black arrows indicate outer segment-like structures while white arrows indicate inner segment structures. (**e**) Quantitative analysis of select photoreceptor genes (NRL, NR2E3, Rhod, SW-, MW- and LW-opsin, arrestin and the calcium channel proteins CNGA3, and CNGB3) was carried out to show quantifiable changes in gene expression. Target genes were normalized against the geometric means of the reference housekeeping genes CREBBP, FBXL12, and SRP72. Data reflects the expression levels from pooled samples (n = 10 per time point) from three replicates per time point and error bars represented as s.e.m. The difference between the indicated time points is statistically significant by Student’s t-test where ****P < 0.0001, ***P < 0.001, **P < 0.01, *P < 0.05; ns, not significant. POS = photoreceptor outer segment; PR = photoreceptors; RC = retina cup. Scale bars = 50 μm.

**Figure 6:**
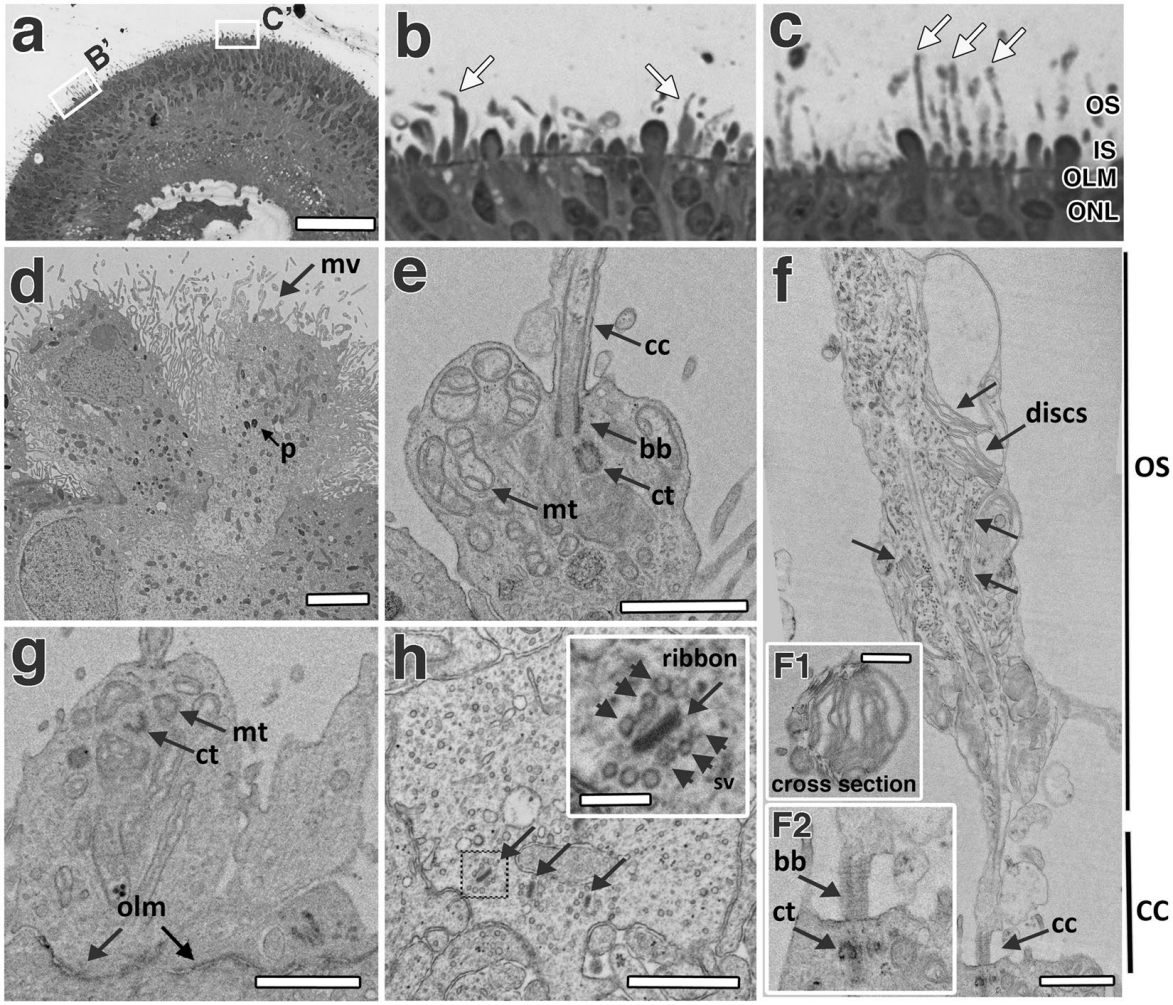
Electron micrographs of EP1-iPSCs derived retina cups at 160 days. (**a**) A one-micron thick section of a plastic embedded retina cup. Highlighted boxes (B’ and C’) are represented in panels (**b**) and (**c**) respectively. Arrows indicated in panel (**b**) represent cones while those in (**c**) indicate rods. 50 nm ultra-thin sections were imaged by standard EM. (**d**) Microvilli (mv) present on the surface of isolated RPE clumps RPE and pigment (**p**) within; (**e**) a PR inner segment with a connecting cilium (cc), mitochondria (mt), a centriole (ct), and basal body (bb); (**f**) a PR with a connecting cilium bearing a rudimentary outer segment with stacks of discs (arrows). (**F1**) Panel illustrates a cross section of a POS bearing organized discs while (**F2**) shows the basal body and centriole; (**g**) a PR inner segment with an intact outer limiting membrane; (**h**) a PR synaptic terminal with synaptic vesicles (arrowheads) docked at electron dense ribbons (arrows). PR = photoreceptor, RC = retina cup; RPE = retinal pigment epithelium. Scale bars (**a**) = 300 μm, (**d**) = 4 μm, (**e**) = 1 μm, (**f**) = 1 μm, F1 = 500 nm, (**g**) = 1 μm, (**h**) (inset) = 200 nm, (**h**) = 1 μm.

**Figure 7:**
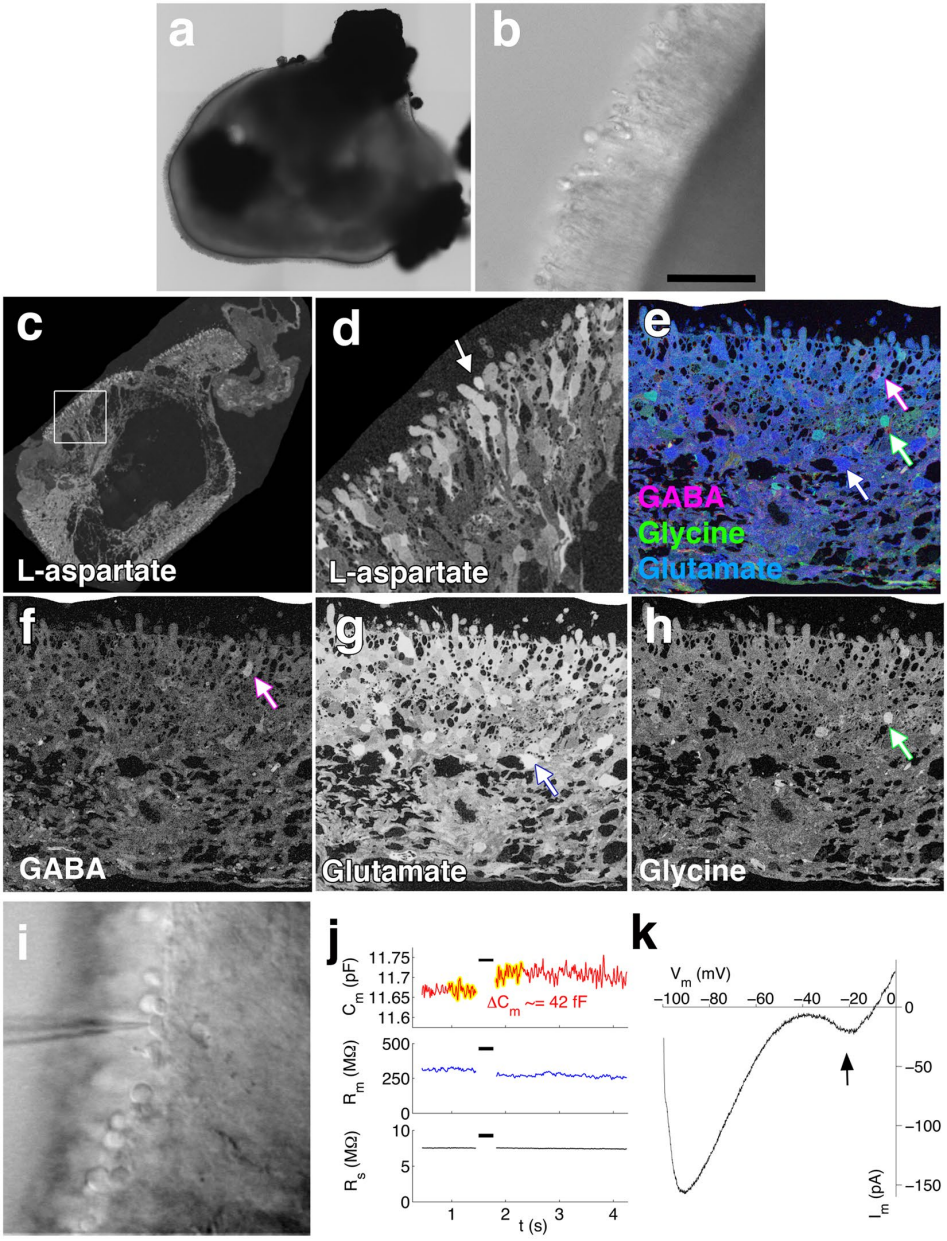
Metabolic labeling and electrophysiology of retina cups. Brightfield image of D300 IMR90.4 derived RC shown at (**a**) low and (**b**) high magnification. Immunolabeling of ultrathin plastic sections of RCs with antibodies against (**c**,**d**) L-aspartate, (**e**,**f**) GABA, (**e**,**g**) Glutamate and (**e**,**h**) Glycine on D300 RCs. Panel d is a higher magnification of the inset box from (**c**). Panel e is a triple label of GABA, glutamate and glycine. A DIC image of a RC in panel (**i**) illustrates a recording pipette used for capacitance measurements. (**j**) Upper panel: Lock-in membrane capacitance measurement of a cone photoreceptor from a PSC derived eyecup showed a capacitance “jump” upon a brief depolarization (indicated by the black bar) from −60 mV to −10 mV. The yellow highlights are regions before and after the stimulation from which the capacitance values are averaged. Middle and lower panels show stable membrane resistance and series resistance during the course of the recording, suggesting that the capacitance jump in the upper panel is not an artifact of conductance change, rather it reflects synaptic vesicle release. (**k**) In a whole cell recording of a cone photoreceptor, the voltage was held at −60 mV, then stepped to −100 mV, and increased to 0 mV at a ramp speed of 100 mV/250 ms (0.4 mV/ms). The arrow points to the peak calcium current at −20 mV, which is typical of L-type calcium currents in mammalian photoreceptors. Scale bars b = 40 μm.

A number of published protocols describe methods to generate optic vesicles with retina cup-like structures^6, 14, 16–18, 27^. We sought to build upon the generality, reproducibility, and simplicity of existing 3D culture protocols to develop conditions that promote mature photoreceptor outer segment (POS) structures. To ensure that OV formation was not cell line-specific, we worked in parallel with three hPSC lines, the IMR90.4 and EP1 iPSC lines (Figs 1–7) and the H7 ESC line (Supplemental Fig. 3). Since, the generation of moderate sized OVs from serum-free embryoid body aggregates (SFEBs) using the protocol described by Nakano *et al*.^16, 17^ takes 17 days, we first worked to optimize the vesicle generation step. Based on the hypothesis that growth medium composition, O_2_ concentration, and aggregate size could each impact cell differentiation, we systematically optimized each of those parameters.

Knockout serum replacement (KSR), has been cited as promoting caudal features^17^, so we first eliminated that from our neural induction medium (NIM). We tested E6 supplement (similar to previously published E8 stem cell medium supplement minus TGF-β1 and FGF2)^28^ and observed that E6 alone produced highly folded neural vesicles but was inadequate for obtaining organized optic vesicles; doubling the E6 concentration qualitatively enhanced vesicle morphology and the addition of B27 (-VitA) to make “BE6.2” medium further enhanced organized 3D neural vesicle formation. During the first week following aggregation, cells grown in KSR or BE6.2 developed into similarly sized aggregates but differed in that day 5 (D5) KSR treated samples were more compact with no vesicles while BE6.2 medium supported some vesicle formation (Fig. 1b; arrow). With the new optimized medium we observed that during the first 6–12 hours, dissociated cells coalesced into uniform spheres at the base of the U-bottom wells (Supplemental online Video 1). The majority of the cells integrated into intact spheres; however, a small halo of dead cells (Fig. 1d; white arrow) often persisted for several days. Unlike the aggregates described by Nakano *et al*.^17^ grown in V-bottom plates, our protocol utilized U-bottom plates and a slow gravity-based aggregation. U-bottom plates, in addition to working well for formation of consistent aggregates, also have the advantage that they are better suited for imaging.

Since hypoxia can improve the pluripotency and proliferation of hPSCs^29–31^ and blebbistatin can increase cell survival when passaged to single cell levels^32^, we reasoned that recovery under these conditions, might improve aggregate formation. Relative to aggregates initiated outright in 20% O_2_ (normoxia), aggregates maintained in 5% O_2_ (hypoxia) for 1 additional day demonstrated increased viability, and by D8 had more vesicles per sphere and were larger in size (Fig. 1e, Supplemental Fig. 2). Their average sizes were statistically greater (n = 15 per data point; p < 0.05), in 5% O_2_ (Fig. 1e). By tracking an individual vesicle within the same SFEB (Fig. 1d; arrows) from D6-10, vesicle numbers appeared constant and vesicles grew in size (Fig. 1d). We next optimized initial SFEB size by titrating cell numbers from 1,000 to 9,000 cells per well. Those originating from 1,000 cells remained smaller and yielded less morphologically distinct vesicles while those initiated with 9,000 cells initially grew well but by D10 appeared overgrown with dark necrotic cores. For all three lines (IMR90.4, EP1 and H7), plating 3,000 cells appeared optimal for producing neural vesicles with the widest possible range of time during which vesicles could be easily discriminated.

Mechanically isolated optic vesicles were grown as indicated (Fig. 2a). In early stages during the forced aggregate and optic vesicle stages, pluripotency expression (e.g. *OCT4*) was reduced (Fig. 2b) and early neural retina genes became expressed (e.g. *SIX6*; Fig. 2o). Mechanically excised from the SFEB’s (Supplemental Fig. 3), vesicles were maintained at low density since at high density they often coalesced into ‘caterpillar-like’ chains with necrotic cores. Poor quality vesicles with an opaque appearance or with signs of necrosis were regularly discarded. In addition, vesicles that were insufficiently excised at D12 were further trimmed to approximately 500 microns in size to prevent overgrowth in subsequent weeks.

### Isolated optic vesicles give rise to laminated 3D retina cups

After 1 month of periodic grooming (removal of non-RC like structures and trimming of overgrown vesicle), 3D translucent RCs appeared relatively homogeneous, with minor differences in shape and size (Fig. 2c–e). It was not uncommon to see a gradual loss of high quality 3D cups over the first two months, suggesting that either some of the OVs, previously identified on the basis of morphology, were not retinas in the first place or that conditions for maintenance were suboptimal (Supplemental Fig. 4d,e). At D35, OTX2+ cells were scattered throughout the retina with a higher concentration near the presumptive outer retina where PRs reside (Fig. 2f–h), suggesting development of an increasingly organized retina. On the opposite side of the retina, BRN3+ retinal ganglion cells (RGCs) (Fig. 2i–k) were present. While these were abundant at early time points, BRN3+ cells were progressively lost and were not detectable at later stages (D160). At D35, NF200+ staining was present throughout the retina with a greater distribution at the ganglion cell border where RGC axons are expected to reside (Fig. 2l–n). When analyzed by quantitative PCR, *SIX6* and *OTX2* had patterns of expression that were elevated over time (Fig. 2o,p). *BRN3B*, by contrast, peaked around D65 and became markedly decreased thereafter, presumably reflecting a loss of RGCs (Fig. 2q).

At D45 RCs had a translucent appearance (Fig. 3a) and recoverin (RCVN) + cells were detected along the outer RC where the prospective outer nuclear layer (ONL) resides (Fig. 3b,c). *VSX2* mRNA was detected at D30, peaked at D65 and stabilized thereafter (Fig. 3d). *RCVN* mRNA expression was detected by D30, with a dramatic increase by D65 (Fig. 3e). Following this initial spike, *RCVN* leveled off by D100 and remained fairly constant thereafter. *CRX* (Fig. 3f) became elevated at D65 and continued to rise with time. In addition to showing laminar organization of their inner retina-like region, the RCs also showed organized PAX6+ neurons that localized to the inner retina, including the inner nuclear layer (Fig. 3h). RCs at late stages showed structures resembling photoreceptor inner and outer segments (PIS/POS; Fig. 3g). It should be noted that although RCVRN+ cells were generally located along the outer aspect of the RCs, variability in the position of these cells did exist with some cells even located deep within the RC.

### Synaptic ribbons form in long-term retinal photoreceptors

Synaptic ribbons, found in mature rod spherules and cone pedicles, are essential for retinal function. In the outer plexiform layer (OPL) ribbons form a tripartate junction with bipolar and horizontal cell dendrites. To identify these structure in RCs, we performed IHC for Post-synaptic density-95 (PSD95) and C-terminal binding protein (CtBP2) (Fig. 3i–l); the CtBP2 antibody recognizes two isoforms including a transcriptional repressor and a synaptic protein (RIBEYE). At D120, PSD95 was evenly distributed in cells of the outer retina where PRs reside (Fig. 3i). This pattern is unlike the mature retina in which the protein is restricted to the synaptic terminal. CtBP2/RIBEYE at D160 appeared throughout a loosely organized OPL (Fig. 3j–l). In some cases, obliquely oriented ring structures lined with ribbons were present in the presumptive OPL; a feature similar to cone terminals of other species^33^. Peanut agglutinin lectin (PNAL), a marker for cone inner/outer segments and synapses, was abundant in both structures. When combined with RIBEYE labeling, we observed a close association between PR terminals and ribbon staining (Fig. 3l; arrow). Interestingly, while inner/outer segments were considerably more organized, the OPL was less organized. Outside of the presumptive OPL, RIBEYE was less abundant and synaptic lamination was less organized. Whether the bipolar cells expressing RIBEYE failed to form properly in the first place, or became disorganized during degeneration related remodeling, is unknown. The lack of BRN3 at later stages suggests that RGC loss might be a function of or a cause of the observed inner retina disorganization.

### Rod and cones become developmentally advanced late during RC development

All-trans retinoic acid (ATRA) has well documented effects on retinal development^2–4, 34, 35^. While necessary for development, its continued presence can hamper maturation^17^. Based on this, we treated RCs with 500 nM ATRA from 20 days (after retina formation) until 120 days (before PR maturation). Shortly thereafter, small sprouts began to emerge from the RC surface, and by D160 POS-like structures were present (Figs 3g, 4a, 5b and 6a–c, Supplemental Fig. 5). Variability in the length and onset of OS formation was observed (Supplemental Fig. 5). Inner/outer segment growth was self-limiting, reaching a terminal length of approximately 39 μm, a range similar to that reported *in vivo* ^36–38^. Unlike the cone rich fovea *in vivo*, rhodopsin + rods and M/L opsin+ cones in RCs were generally evenly dispersed across the mini-retinas (Figs 4c–e and 5c,d). RPE also frequently grew on RCs opposite to the retina (Figs 4a and 7a), or as independent spheroids (Fig. 4f–h), with honeycomb shaped polygonal morphologies (Fig. 4g,h). Interestingly, long POS structures developed, even without an adjoining RPE, suggesting that, at least *in vitro*, POS’s don’t require direct cell-cell contact with RPE for formation or maintenance. These RPE cells, however, might still provide diffusible factors capable of influencing retinal development. After D200 (W28), the POS layer was denser (Fig. 5b) and at D300, visual pigments were compartmentalized to the POS (Fig. 5c,d; dark arrows), as opposed to the cell soma. To a lesser extent RHOD and R/G OPSIN signals were occasionally present within inner segments (Fig. 5c,d; white arrows). Quantitative PCR was used to evaluate the expression profiles of genes expressed in rod and cone photoreceptors (Fig. 5e). The rod PR genes *NRL*, *NR2E3* and *RHOD* were each expressed at D100 and beyond. Short wavelength opsin (*SW*-*OPSIN*) showed an almost 7-fold increase by D65, while medium and long wavelength opsins (MW- and LW- opsin) were detected abundantly only after D100. To measure whether visual transduction machinery was expressed, we studied *ARRESTIN* (*ARR*) and the calcium-gated channels *CNGA3*, *CNGB3*. While the calcium channels were typically expressed first, all three genes were detected by D100.

### Retina cups recapitulate morphological aspects of the human retina

One-micron plastic sections were used to study outer retina ultrastructure, including inner and outer segment structure. RC’s were organized into discrete nuclear layers (Fig. 6a), with cone-like (Fig. 6b) and rod-like (Fig. 6c) cells populating the prospective ONL. Similar to the *in vivo* situation, cones were shorter, with comparatively larger PISs, and stubbier than rods, which had comparatively smaller PISs. The POSs in 1 micron RC sections (Fig. 6a–c) are more sparse in appearance as compared to immunohistochemical sections (Fig. 4c–e) that are generally much thicker. In 50 nm ultrathin sections, microvilli could be observed extending from the RPE cells (Fig. 6d). In the PIS, near the connecting cilium (cc), we observed basal bodies (bb) and a centriole (ct) with microtubules in a classical ‘9 + 2’ arrangement (Fig. 6e). Between the PIS and POS was a connecting cilium (cc) with microtubule tracks (Fig. 6f). In the presumptive POSs, we saw stacks of discs (Fig. 6f,F1) which although still somewhat immature, are reminiscent of disc formation during mid-stages of rat POS development^39^. An electron dense layer below the inner segment structures corresponds to the outer limiting membrane (OLM) formed by Müller glia protrusions (Fig. 6g). Electron dense horseshoe shaped structures in the presumptive OPL, corresponding to synaptic ribbons, had synaptic vesicles docked at their surface (Fig. 6h).

### Metabolic labeling and functional characterization of retinal neurons

In order to assess the presence of neurotransmitters, which are essential for retinal function and reflect a state of relative retinal maturity, we used metabolic labeling with immuno-EM to detect several critical retinal neurotransmitters^40–43^. At D300, RC’s bore outer segments protruding from their surface (Fig. 7a,b). The excitatory transmitter L-aspartate was found at highest levels in the putative ONL (Fig. 7c,d). Triple labeling for the inhibitory transmitters GABA and glycine and the excitatory transmitter glutamate (Fig. 7e) revealed weak immunoreactivity for GABA throughout the retina (Fig. 7f), widespread expression of glutamate spanning the entire retina (Fig. 7g) and sparse localization of glycine along the outer portion of the inner nuclear layer (Fig. 7h).

Synaptic function of photoreceptors in the eyecup was assessed using whole-cell patch clamp recording to measure total membrane capacitance (Fig. 7i–k). Photoreceptors were clamped at −65 mV and depolarized to −10 mV to trigger vesicle release. Stimulus-evoked “jumps” in capacitance that were not accompanied by simultaneous changes in either the membrane or series resistances were taken as evidence of vesicle exocytosis (see Methods). Of 16 recorded cells, 8 cells exhibited capacitance jumps ranging from approximately 10-40 fF (Fig. 7j). Assuming a single-vesicle capacitance of 0.05 fF^44, 45^, this range of capacitance increase corresponds to the fusion of 200–800 synaptic vesicles. Additionally, a ramp protocol was used to determine the presence of voltage-gated currents. Downward (depolarizing) deflections occurring at approximately −40 mV, which are typical of L-type Ca^2+^ currents, were commonly seen in these cells (14 out of 16 recordings) (Fig. 7k). These recordings also exhibited super-linear depolarizing currents at more hyperpolarized potentials (approximately −90 mV and below), which is a hallmark of the HCN-type current commonly found in cones^46, 47^.

## Discussion

Retinal cells differentiated as 2D monolayers or cell clumps form a heterogeneous mix of retinal and non-retinal cells^2–4, 7, 9, 13, 25, 48, 49^, whereas PSC-derived 3D retinas tend to be more organized^5, 6, 11, 16–18, 27^. Such 3D ‘mini-retinas’ contain each major retinal cell type and become organized into laminated structures with PRs and RGCs populating the outer and inner regions, respectively. These PRs in these 3D retinas emulate many of the temporal and spatial characteristics of *in vivo* development^5, 6, 16–18, 27^. Building on the growing success of those pioneering studies, we placed our emphasis on generating 3D retinas capable of supporting POS outgrowth similar in appearance and structure to that observed *in vivo*.

Many factors can affect PSC differentiation *in vitro*, and there is considerable variation in how cells are grown. Oxygen concentration (hypoxia versus normoxia) and the substrate (feeder layer versus feeder-free) on which stem cells grow are two examples^17, 50^. In this study, we eliminated feeder cells and maintained PSCs in hypoxia as described previously^51^. Under hypoxic conditions, PSC’s are less stressed, divide faster, have less cleaved caspase-3, and exhibit fewer chromosomal abnormalities^30^. Since bactericidal antibiotics can cause mitochondrial dysfunction and reactive oxygen species (ROS) overproduction^52^, we eliminated these from our cultures. Cumulatively, these changes are likely to contribute to the positive growth and differentiation that we observed; however, a more rigorous evaluation across different protocols and labs would be helpful to validate these conditions further.

In the human retina *in vivo*, POS’s emerge as small buds at around W23-25 with elongation occurring at about W32^36, 53, 54^. In our system, POS’s formed between W18-28 (D130-200), which though somewhat precocious is generally within the expected time range. These observations are generally consistent with work from Zhong *et al*.^6^ who showed small outer-segment discs at W28, Parfitt *et al*.^19^ who demonstrated apical cilia emerging from mitochondria-rich inner segments by W13 and outer segments emerging by W21, and work from Lowe *et al*.^15^ who similarly demonstrated outer segment formation at day 187^6, 15, 19^. The dense cilia and visual pigment expression (e.g. RHO, OPN1LW/OPN1MW) coupled with ultrastructural observations at the EM level (e.g. outer limiting membrane, connecting cilium, etc.) are very similar to what we have observed. Such independent observations are reassuring given the considerable technical variation associated with these different methodologies.

Metabolically, the presence of excitatory and inhibitory neurotransmitters is notable. Although GABA, glycine and glutamate are central carbon skeletons in many metabolic processes, the presence of neurons in the RCs that appear to be dominated by glycine and GABA implies further specialization of retinal cell classes. Light responsiveness and synaptic transmission are two key features of functional maturation of photoreceptors. Zhong *et al*. reported light responses from a limited number of photoreceptors from RCs^6^. However, it is not known whether photoreceptors in such RCs have mature synapses. Here we examined the synaptic function of photoreceptors in our RCs using membrane capacitance changes as an index of voltage-dependent synaptic vesicle release. In half of the cells recorded, we observed exocytosis upon depolarization, and the range of capacitance jumps observed here is very similar to that seen in mature ground squirrel photoreceptors (our unpublished observations as well as those reported by Grabner *et al*.^55–61^). In addition, the voltage-dependence of voltage-gated currents, such as the putative Ca^2+^ currents and HCN currents, are very similar to what have been reported for mature photoreceptors. Taken together, it appears that these cells have synaptic machinery that are capable of forming functional synapses.

The role of RPE in promoting and maintaining POS’s is complex. Some studies suggest that RPE contact is necessary for development and survival of PRs^55–61^. Neurospheres maintained in RPE conditioned medium, for instance, had improved lamination^62, 63^. Our own RC’s contain RPE patches and could benefit from such diffusible factors. That we saw nearly full length POS’s suggests that cell-cell contact with RPE is not critical for basic development and/or maintenance of POS *in vitro*. This is contrary to *in vivo* retinal detachment models in which PRs are dysfunctional and eventually die in the absence of direct RPE contact^64, 65^. Since RC’s are not exposed to *11*-cis retinal, a necessary component for phototransduction, one possibility might be that they may be less physiologically active and thus less susceptible to injury^66^.

Despite the achievements of this current study, batch-to-batch variation still remains an issue and manual selection is still necessary. Also, in late stages of development, the inner retina appears disorganized, suggesting a failure to form normally or disorganization with time. A lack of BRN3+ RGCs in late stage retinas supports the possibility of ongoing degeneration during culture and warrants further attention. Adding neuroprotective compounds to support RGC health and survival could be helpful in this regard^67^. In fact, it may be possible to take advantage of the observed death of RGCs to develop an assay for RGC survival-promoting molecules. In addition, one new approach demonstrated that early in development BMP4 can increase the number of RAX+ 3D retinas^50^. Later in development, blocking BMP and TGF-β signaling enriched the number of cones that developed^68^. It is possible that modulating BMP4 and other diffusible signaling molecules will further increase the efficiency of our system too.

## Methods

A detailed supplemental cell culture protocol is available for download. Antibodies are described in Supplemental Table 1.

### Cells

Line IMR90.4 iPSCs^69^ and line H7 (WA07) (WiCell) and the episomal derived line EP1.1 iPSC^10^ were used. Each was used for early RC studies, while only IMR90.4 and EP1 cells were also used for late stage experiments. Cells were routinely tested for mycoplasma by PCR^70^. Pluripotency was evaluated with antibodies for NANOG, OCT4, SOX2, SSEA4 (Fig. 1a).

### Stem cell maintenance

Stem cells were routinely maintained in either mTeSR1 or Essential 8 (E8). However, for all cell aggregation experiments, cells were maintained on 1% (vol/vol) Matrigel-GFR™ (BD Biosciences) coated dishes at 37 °C under hypoxic conditions (10% CO_2_/5%O_2_) in mTeSR1 (Stem Cell Technologies) prior to reaggregation^28, 51, 71^. Cells were passaged with Accutase (Sigma) for 8–10 minutes, dissociated to single cells, quenched with mTeSR1 plus 5 μM blebbistatin (B; Sigma), pelleted at 80 × g for 5 minutes, resuspended in mTeSR1 + B and plated at 5,000 cells per 35 mm well^32^. After 48 hours, cells were fed without B. To minimize cell stress, no antibiotics were used^52^.

### Cell culture medium

E8 (E6 supplement plus FGF2 and TGFβ1) medium for cell maintenance consists of DMEM/F12 (1:1) (#11330-032; Invitrogen) with 19.4 mg/L insulin (#11376497001; Roche), 64 mg/L L-ascorbic acid (#A8960; Sigma), 14 μg/L sodium selenium (#S5261; Sigma), 10.7 mg/L transferrin (#T0665; Sigma), 19.4 mg/L NaHCO3, 100 μg/L FGF2 (#AF-100-18B; Peprotech), 2 μg/L TGFβ1 (#100-21; Peprotech). Osmolarity was raised +30 mOsm to ~330–340 mOsm by adding 0.88 g/L NaCl^28, 51^. BE6.2-NIM (B27 + E6 at 2X concentration) (neural induction medium) for cell differentiation consists of DMEM (#11965; Invitrogen) with 1% B27 vitamin A (−) (#12587010; Invitrogen) and a 2X concentration of E6 supplement (E8 minus FGF2 and TGFβ1). Osmolarity was raised as above. LTR (Long-Term Retina) medium was a 3:1 mix of DMEM (Invitrogen #11965): F12 (#11765: Invitrogen) with 1% B27 (#17504044: Invitrogen), 10% heat inactivated FBS (#16140071; Invitrogen), 1 mM pyruvate (#11360; Invitrogen), 1xNEAA (#11140, Invitrogen), 1X Glutamax (#35050061; Invitrogen) and 1 mM taurine (#T-8691; Sigma).

### Optic vesicle and long-term differentiation of retina cups

PSCs were maintained in mTeSR1 prior to differentiation. High quality PSC’s with minimal spontaneous differentiation were used to initiate serum-free embryoid bodies (SFEBs). Cells were passaged with a longer Accutase treatment (12 minutes) and 3,000 cells in 50 μl’s of mTeSR1 + B were seeded per well into 96-well ultra-low adhesion round bottom plates (#AU96; NOF America). These were differentiated by 2 approaches; the first used Knockout serum-replacement (KSR; Invitrogen #10828-028) as described by Nakano *et al*.^26^; the second utilized BE6.2. In both cases, aggregates formed overnight by gravity. To maximize survival, cells were maintained for one day in hypoxia (10% CO_2_/5% O_2_) then transferred to normoxia (5% CO_2_/20% O_2_). Over the first 4 days (D0-4), aggregates were transitioned to KSR or BE6.2 medium adding 50 μl fresh volume daily. On D4-8 a 50% medium exchange was performed daily and every other day thereafter. To promote an anterior neural fate, medium contained 3 μM Wnt inhibitor (IWR1e; #681669; EMD Millipore) from D1-6 and 1% (v/v) Matrigel from D1-8. Aggregates were transferred at D10 to 15 ml tubes, rinsed 3x in HBSS, and suspended in BE6.2 + 100 nM Smoothened agonist (SAG; #566660; EMD Millipore) from D10-D12 to enhance retinal induction then LTR + SAG from D12-D18. Using sharpened tungsten needles (see Supplemental Fig. 1 and Supplemental online Video 2), vesicles were typically excised from D10-12 (as late as D16) and fed every 2–3 days in suspension in ultra-low binding T-75 flasks (Corning) or untreated 10 cm polystyrene petri dishes. When 10 cm dishes were used, sedentary aggregates were monitored to ensure that they didn’t stick to the surface. RCs, identified by morphology, were maintained at 37 °C in standard 20% O_2_/5% CO_2_. To increase survival and differentiation, 500 nM all-trans retinoic acid (ATRA; #R2625; Sigma) was added to LTR medium from D20 until D120. 10 μM Gamma-secretase inhibitor (DAPT; #565770; EMD Millipore), which blocks notch signaling and is thought to promote PR generation at later stages, was used from D29-45. RCs were grown at low density (30–50 per 10 cm dish) to reduce aggregation.

### Measurements and statistics

SFEBs (n = 15) in 5% and 20% O_2_ (Fig. 1c–e) were measured at D1-6 along their radial diameter. Average diameters (in microns) were plotted as a function of days in culture using Prism6 software (n = 15 per time point) and statistical significance determined using multiple t-tests using the Holm-Sidak method with an alpha cutoff of 0.05 for statistical significance (Fig. 1e). Nikon Elements Software was used to capture and measure inner/outer segment lengths at D300 from RC’s bearing the longest POS-like structures. 17 measurements were taken to calculate IS/OS length.

### Fixation and immunohistochemistry (IHC)

Adherent cells were fixed on ice in 4% PFA in 0.1 M PB-5% sucrose for 5 minutes; floating OVs and RCs were fixed for 25 minutes. On ice, RCs were immersed sequentially in 6.75% and 12.5% sucrose-PBS for 1 hour each, 25% sucrose-PBS overnight, 1 hour in a 2:1 ratio of 25% sucrose-PBS/OCT Tissue-Tek (Ted Pella), and snap-frozen on dry ice. 8 μm sections were mounted onto Superfrost Plus slides (ThermoFisher) and incubated overnight in primary antibodies in 2% normal donkey serum (NDS) and 0.1–0.2% Triton X-100 in PBS. Secondary antibodies were anti-mouse, -sheep and -rabbit IgG’s (H + L) coupled to Alexafluor-488, −546, or −647 (Invitrogen, 1:1,000). 10 μg/ml Hoechst 3342 (Molecular Probes) was used to visualize cell nuclei. Sections processed without primary antibody were used as controls. For whole-mount IHC, 3D RCs (Fig. 4) were blocked and permeabilized for 1 hour in 10% NDS, 0.2% Triton X-100 (TX100) in PBS, then incubated with primary antibodies against the visual pigments in PBS containing 2% NDS and 0.2% TX100, rinsed in PBS, and visualized with secondary antibodies (as above).

#### Microscopy and image processing

Images were acquired with a NIKON TE2000 or LSM710 laser scanning confocal microscope. Confocal microscopy was performed with similar settings for laser power, photomultiplier gain and offset, with a pinhole diameter of one Airey unit. Thin optical sections were used for subcellular localization or co-localization (Figs 2–5). Images were adjusted for brightness and contrast using ImageJ (NIH; http://rsb.info.nih.gov/ij/) or Adobe Photoshop. Maximum intensity projection z-stacks (5–10 optical sections, 0.5–1.0 μm thickness, 0.3 μm step size) were rendered to give a more inclusive picture within the tissue sections.

### Electron microscopy (EM)

RCs were fixed in cold 2.5% glutaraldehyde/2% PFA phosphate buffer, in 1% osmium tetroxide, dehydrated and embedded in Eponate. 50 nm ultra-thin sections were cut and stained with uranyl acetate and lead citrate and imaged using transmission electron microscopy (Hitachi H7600).

### Computational Molecular Phenotyping (CMP)

Samples were fixed in 1% PFA/2.5% GA in 0.1PB containing 3% sucrose and 1 mM MgSO_4_, followed by dehydration in graded methanol and acetone, embedded in Eponate and cut to a 90 nm thickness on a Leica Ultracut microtome. Serial sections were probed using antibodies targeting small molecules L-aspartate, L-glutamate, glycine, L-glutamine, γ-aminobutyric acid (GABA). Antibodies incubated overnight at room temperature were visualized with goat anti-rabbit secondary IgG coated with 1.4 nm gold (Nanoprobes Nanogold® -anti Rabbit IgG) and silver intensified for CMP^41^. 8-bit images (243 nm/pixel) were mosaicked and registered with ir-tweak (https://www.sci.utah.edu/download/ncrtoolset.html)^40^. RGB channels for display images were linearly contrast stretched for display. Molecular signals were visualized as rgb maps (e.g., γGE → rgb assigns γ-aminobutyric acid, glycine and L-glutamate to red, green, and blue color channels, respectively). For display only, raw data channels were linearly contrast-stretched and sharpened with unsharp masking. Monochrome and RGC images were intensity mapped.

### Functional recording of photoreceptors

Individual cells were recorded using whole-cell patch clamp to assess membrane currents and exocytosis. Specifically, thick-wall borosilicate pipettes were pulled to 5–10 MΩ tips using a programmable pipette puller (model P-97, Sutter, Novato, CA) and wrapped with parafilm to reduce stray capacitance. Pipettes were filled with intracellular solution containing (in mM) 120 CsMs, 6 MgCl, 4 ATP-Na_2_, 1 GTP-Na, 0.5 EGTA, 20 HEPES. Cells were continuously perfused at room temperature (22 °C) with extracellular solution containing Ames’ medium (Sigma-Aldrich, St. Louis, Mo), oxygenated with a 95% O_2_/5% CO_2_ air mixture, and buffered with sodium bicarbonate to achieve a steady pH of approximately 7.35. Recording was performed using an EPC USB 10 double amplifier and PATCHMASTER software v.2 × 73 (HEKA, Holliston, MA).

Exocytosis was assessed using the lock-in feature of the EPC amplifier in the “Sine + DC” mode, which provides continuous estimates of membrane capacitance (C_m_), resistance (R_m_), and series resistance (R_s_). In this protocol, the cell membrane potential was modulated using a 15 mV, 1 kHz sine wave centered around −65 mV. This sine wave was briefly interrupted by short (ranging from 10–500 ms) depolarizations to −10 mV (“stimulation”) to evoke exocytosis. The resulting C_m_ traces were digitally low-pass filtered at 20 Hz to allow the measurement of “jumps” in baseline capacitance due to this exocytosis. Because C_m_ traces exhibited slow baseline drifts, they were also slope-corrected using the average slope of the trace during the 1-s period prior to stimulation. Membrane currents were broadly assessed using a ramp protocol. From a holding potential of −65 mV, cells were hyperpolarized to −100 mV and increased to 0 mV over 250 ms (0.4 mV/ms). Voltage-gated calcium and HCN-type currents were reflected by depolarizing (downward) current deflections at approximately −95 mV and −40 mV, respectively.

### RNA extraction, cDNA synthesis and quantitative real-time RT-PCR (qPCR) analysis

DNAse I treated RNAs from 10–12 pooled RCs per time point were harvested for total RNA isolation in RLT buffer containing 2% beta-mercaptoethanol using the RNeasy Mini Kit (Qiagen #74104), and resuspended in nuclease free water. RNA concentration and OD260/280 ratio were determined using a Nanodrop 1000 (Thermo-Scientific). Reverse transcription of 1 µg of RNA was carried out using the High Capacity cDNA kit (#4368814; Applied Biosystems) for 10 minutes at 25 °C, 2 hours at 37 °C and 5 minutes at 85 °C. A no RNA negative control reaction was run parallel. For qPCR, we used a 1:30 dilution of the synthesized cDNA. Oligonucleotide sequences are listed in Supplemental Table 2. qPCR was carried out using SsoAdvanced Polymerase (Biorad) and samples normalized using the geometric means of the reference genes CREBBP, FBXL12, and SRP72 as described^12^. Relative normalized expression (delta-delta ct approach) and the standard error of mean (s.e.m) were calculated using Bio-Rad’s integrated CFX manager and Biogazelle’s qbase+ software. Statistical significance between the indicated time points in Figs 2, 3 and 5 was studied by the Student’s t-test where ****P < 0.0001, ***P < 0.001, **P < 0.01, *P < 0.05; ns, not significant.

## Electronic supplementary material

Supplemental Video 1

Supplemental Video 2

Supplemental Info
