# Supplementary material for: Photoreceptor Outer Segment-like Structures in Long-Term 3D Retinas from Human Pluripotent Stem Cells
Source: Sci Rep. 2017 Apr 10;7:766. doi: 10.1038/s41598-017-00774-9 (PMC5429674; doi:10.1038/s41598-017-00774-9)
Supplement: Supplementary file 3 — Supplemental Info [file 41598_2017_774_MOESM3_ESM.pdf]

Photoreceptor Outer Segment-like Structures in Long-Term 3D Retinas from Human Pluripotent Stem Cells.

Karl J. Wahlin[a,b], Julien A. Maruotti[a,c], Srinivasa R. Sripathi[a], John Ball [d], Juan Angueyra [d], Catherine Kim[a], Rhonda Grebe[a], Wei Li[d], Bryan W. Jones[e], Donald J. Zack [a,f].

[a]Wilmer Eye Institute, [b]Shiley Eye Institute, University of California San Diego, La Jolla, California, [c]current address is PhenoCell, Evry, France, [d]Retinal Neurophysiology Section, National Eye Institute, Bethesda, MD, [e]Moran Eye Center, University of Utah, Salt Lake City, Utah, and [f]Department of Molecular Biology and Genetics, Neuroscience, and Institute of Genetic Medicine, Johns Hopkins University School of Medicine, Baltimore, Maryland.

Supplemental online Video 1. Video demonstrating the initiation of forced aggregates over 24 hours. Aided by gravity, stem cells dissociated into a single cell suspension quickly form single uniform shaped spheres at the base of each well in round bottom 96 well plates.

Supplemental online Video 2. Video demonstrating the process for mechanical isolation of neural vesicles. The process for mechanically isolating individual vesicles corresponding to retinal or non-retinal neural tissues is accomplished using electrolytic sharpened tungsten needles in a scissoring motion.

Supplemental Figure 1.

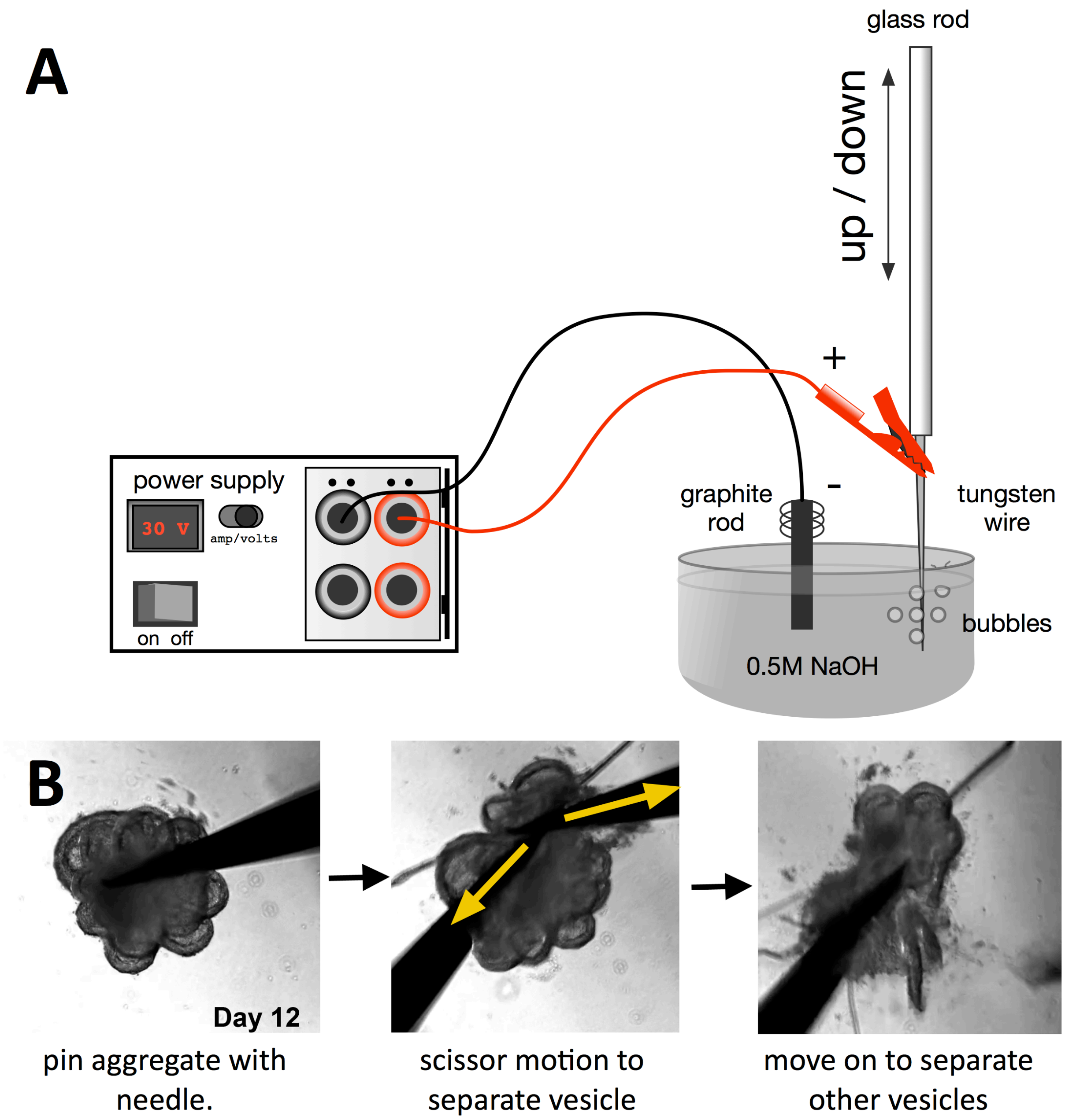

Supplemental Figure 1. (A) Method for electrolytically sharpening tungsten wire (needles) used for excising neural vesicles. Important: Use protective eye wear and perform procedure in fume hood.

Tungsten needles are made by embedding 0.64mm diameter tungsten wire (http://www.emsdiasum.com; cat#73802) into a hollow glass rod using epoxy. To a gel electrophoresis DC power supply, connect the tungsten needle to the cathode and a carbon rod to the anode. Turn on power supply to low (~ 30volts) and immerse both cathode and anode into a 0.5M NaOH solution. Bubbles will form at the tip of the tungsten wire. Repeatedly immerse the tungsten needles into the solution to make a very fine point. For new blunt needles it may be helpful to grind this tip down to a reasonably sharp tip with a sharpening stone or rotating grinder. Once the needle is already reasonably sharp, however, it can be re-sharpened quickly (10-15sec). Sharpening is recommended prior to each cutting session. (B) Visual representation of the scissoring motion used to separate vesicles from larger forced aggregate structures.

Supplemental Figure 2.

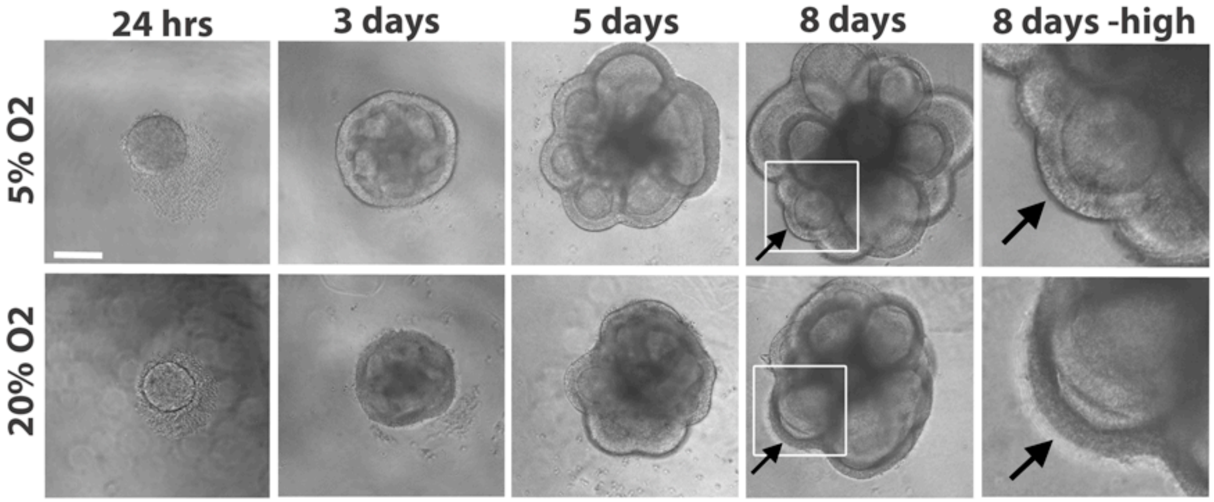

Supplemental Figure 2. Representative images of IMR90.4 derived forced aggregates at 1, 3, 5 and 8 days recovered during the forced aggregate stage at 5 or 20% O_2 for 1 day. Arrows represent elaboration of neural vesicles. Forced aggregates maintained in 5% O_2 (hypoxia) for 1 additional day had increased viability, more vesicles per sphere, and were larger in size at D8. Scale bars =300μm.

Supplemental Figure 3.

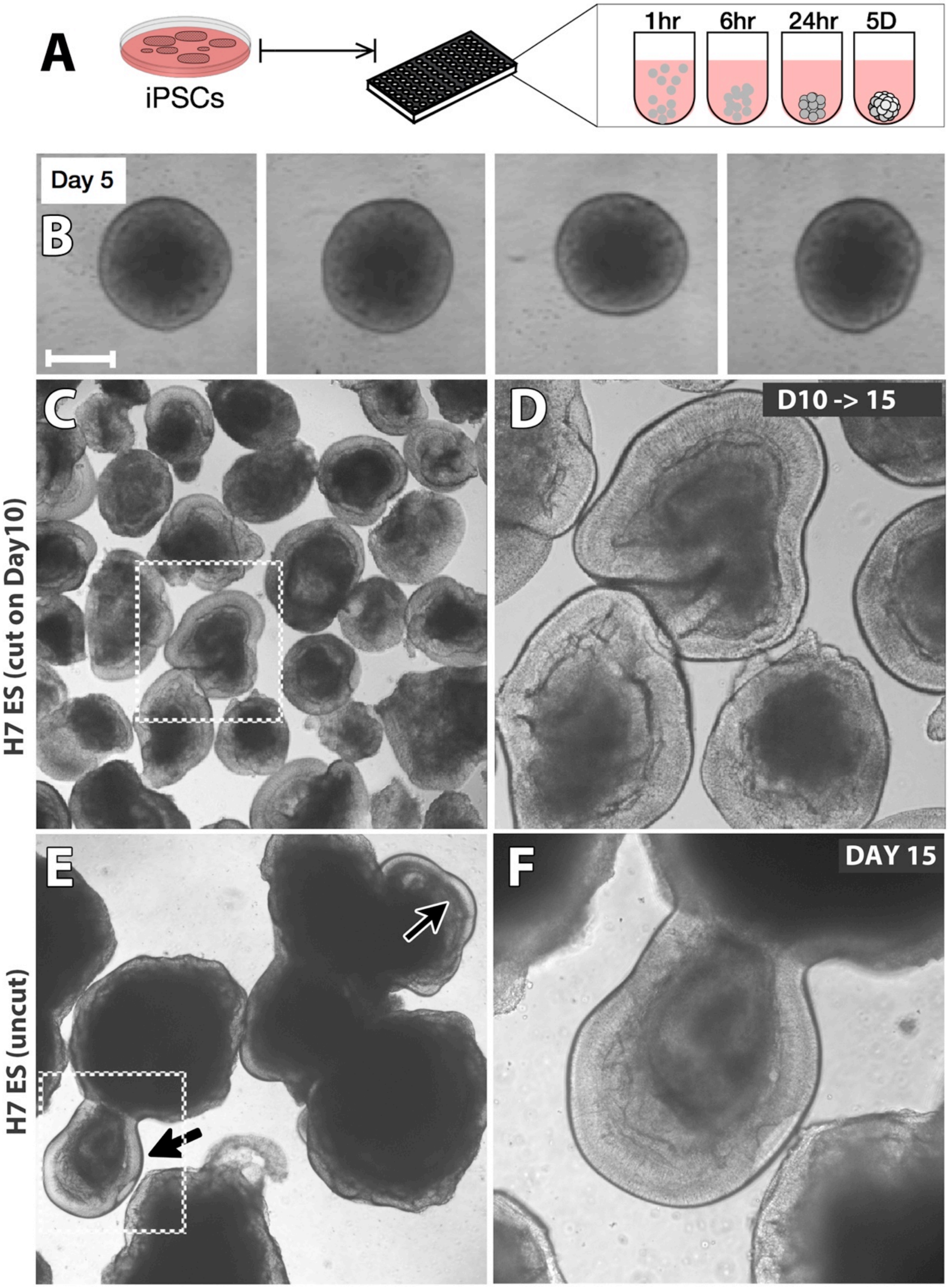

Supplemental Figure 3. When 3,000 cells are seeded into individual wells in a U-shaped round bottom 96 well plate, aggregates assemble within hours into a single uniform sphere at the base of each well that at D5 is highly similar to samples in other wells. Scale bars 500µm in B. (C-F) Neural vesicles resembling early RCs derived from the H7 ESC line. Using sharped tungsten needles, neural vesicles were mechanically isolated from forced aggregates on D10 and observed on D15 (C-D). Optic vesicle structures from uncut aggregates (E-F) at D15 were also readily identified along their surface, however, their numbers were greatly reduced. The sparse number of such vesicles was easier to identify since they tended to have shapes and sizes that were easy to identify and mechanically isolate. Scale bar= 300µm.

Supplemental Figure 4.

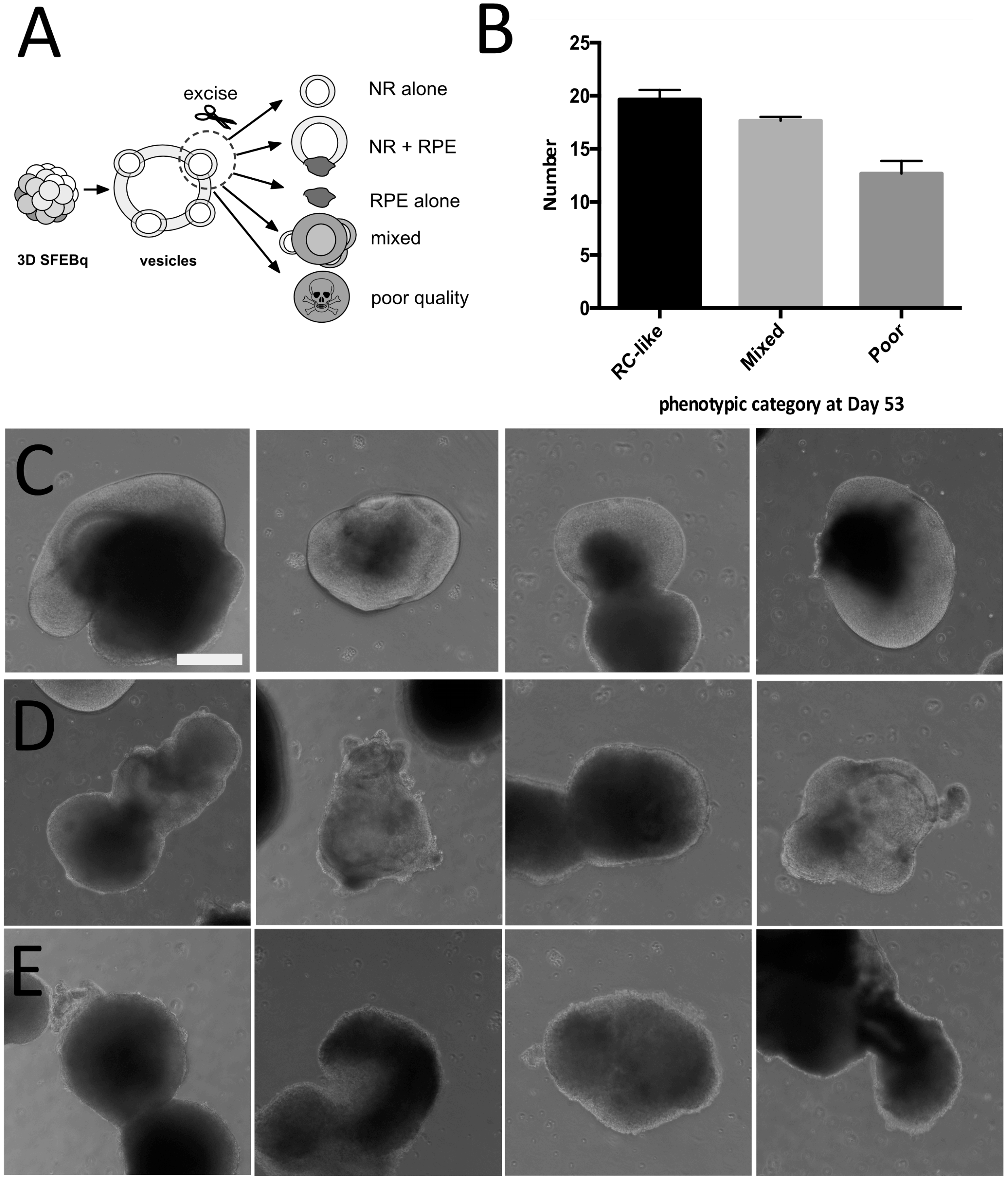

Supplemental Figure 4. Enrichment of EP1 iPSC derived retina cup-like structures after 53 days. (A) Neural vesicles isolated from forced aggregate structures can give rise to a number of phenotypically unique structures including neural retina (NR) alone, NR plus retinal pigment epithelium (RPE), RPE alone, mixed tissue that appears neuronal but is difficult to identify by morphology, and larger necrotic looking structures that appear to have died or are in the process of dying. (B) Quantitation of these structures is presented in graphical form into RC-like, mixed or poor (n=3; sample size = 50). (C-E) Representative images of 3D vesicles with (C) RC-like morphology, (D) mixed populations that are difficult to identify and (E) poor quality samples with a necrotic appearance. Scale bar= 300µm.

Supplemental Figure 5.

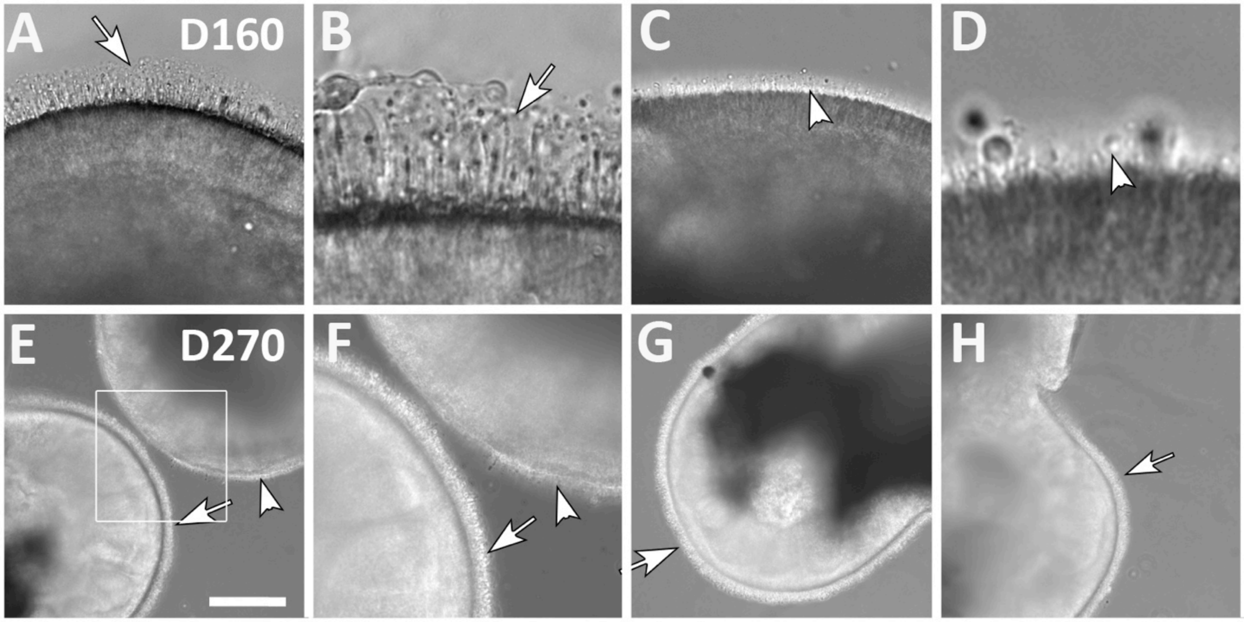

Supplemental Figure 5. Although outer segments frequently form in isolated cups (see arrows), their length can vary significantly. (A,B) shows robust OS-like outgrowth while (C,D) shows minimal outgrowth in age matched D160 samples. At D270 IMR90.4 derived RCs with the most prominent inner/outer segment-like structures are approximately 38.8 µm in length (n=17; SD±4.3). The variability observed at this stage is illustrated by robust outer-segment outgrowth (arrows) and stunted outer segment growth (arrowheads). Scale bar= 300µm.

Human 3D Retinal Cup Formation

In this protocol, optic vesicles can be generated by 12 days. All retinal types, including photoreceptors, develop and form advanced features of outer segment-like structures by 160-200 Days. RPE tissues and anterior neural tissues can also be generated using this approach.

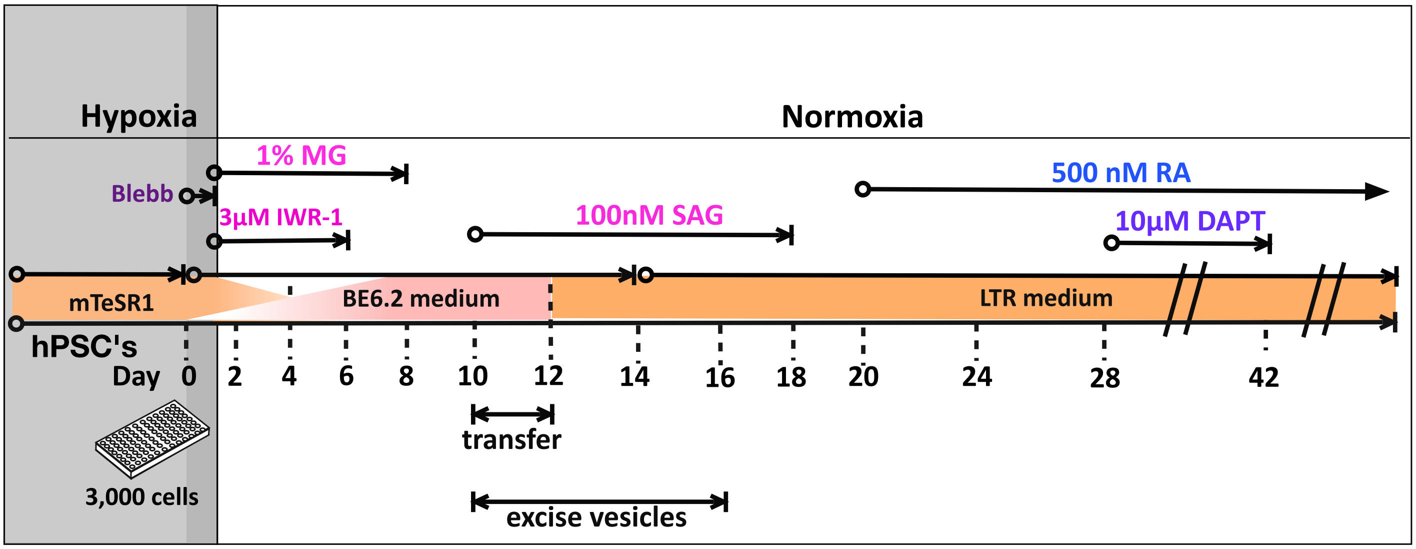

Days 0-8: Forced aggregates - early neural commitment

3-4 days prior to forced aggregation, passage PSC's at 20,000-40,000 PSCs per well (6-well matrigel coated plate).
✪ note - *each 35mm well can produce 300,000 to 1,000,000 cells.*

(1) **Day 0** - To generate SFEBs, treat PSCs with 1ml Accutase for 12 min (longer treatment ensures thorough dissociation), gently triturate up/down 3X with a P1000 pipet, transfer to 2ml mTeSR1 plus 5μM blebbistatin, spin at 80xg for 5 min. then resuspend in 1ml mTeSR1 plus blebbistatin.
(2) add 300,000 cells to 6 ml's of mTeSR1, then add 50μl's (3,000 cells) of mTeSR1+B per well in a non-adherent round (U-bottom) 96-well plate NOF Lipidure). Place back in 5%O_2/10%CO_2 (hypoxia). This gives you 3,000 cells per well.

(3) **Day 1 (24hrs)** - Add 50μl's of **BE6.2** containing 2% (v/v) **matrigel (BE6.2M)** plus 6μM **IWR-1e** Wnt inhibitor. note- add 200μl MG aliquot to 10ml's cold BE6.2.
After 24hrs, **transfer to 20%O_2/5%CO_2 (normoxia) incubator.**
✪✪ BE6.2 medium should be ice-cold when adding **matrigel**, then pre-warmed to 37°C before adding **IWR-1e**.

(4) **Day 2** - Add 50μl's of **BE6.2** + 1%(v/v) **matrigel** + 3μM **IWR-1e.**

(5) **Day 3** - Add 50μl's of **BE6.2** + 1%(v/v) **matrigel** + 3μM **IWR-1e**.

(6) **Days 4-6 - 50% daily exchange** - with 100μl's fresh **BE6.2** + **matrigel** + **IWR-1e**.

(7) **Day 7 - 50% daily exchange** - with 100μl's fresh **BE6.2** + **matrigel**. IWR1e is no longer added.

(8) **Day 8** - Exchange 100μl's of medium again without IWR / Matrigel. Matrigel is no longer added.

Days 8-20: Retinal induction.

(9) **Day 10** - Transfer aggregates to a 15 ml conical tube and rinse 3X in 10ml's of HBSS or BE6.2 medium to remove residual Matrigel and IWR-1e, then transfer to Corning Ultra low binding plates or standard untreated polystyrene 10cm dishes in 10 ml's **BE6.2 medium + 100nM SAG**.
✪ recommended - *limit aggregates to less than 48 aggregates per plate (half of a 96-well plate).*

(10) **Days10-14** - Transfer aggregates to a 35 mm dish and manually isolate optic vesicles using electrolytically sharpened tungsten needles (see supplemental video). Vesicles should ideally be less than 500 microns in diameter since vesicles that are too large tend to overgrow quickly.

✪ note - From D7 - 10 neural vesicles have formed and it may be easiest to excise them from Day 10-12 before they become resorbed into the aggregate. This maximizes the number of vesicles that can be isolated. Vesicles can also be excised for several more days excising only clearly defined vesicles. The later excised vesicles tend to be easier to maintain but are fewer in number.

✪✪ recommended - every few days groom the cultures by removing anything that doesn't look like a 3D OV, separate OV's if they stick together and periodically swirl plates to mix nutrients / metabolic waste.

**Reagents**
**mTeSR1**
- add 5X supplement (-80C) to 400ml's of basal medium. Keep for 2 weeks at 4°C.
**E6 stock medium (50X) - 100ml (10 aliquots)**
- add 36.2ml 7.5% **NaHCO3 (7.5g/100ml)**<br>- add water to 100ml<br>- add 97mg **insulin**<br>- add 53.5mg **holo-transferrin**<br>- add 320mg **L-ascorbic acid** (note- will be cloudy),<br>- add **sodium selenite** (5µl of 1,000,000x [14mg/ml] stock).<br>- Mix thoroughly (*optional*-filter later). Make 10ml aliquots and store at -80°C.
**BE6.2 medium-250ml**
- 10ml **E6** stock<br>- 5.0ml **B27 (-Vit A)**<br>- 2.5 ml **Glutamax** (100X)<br>- 2.5 ml **NEAA** (100X)<br>- 2.5 ml **Pyruvate** (100X)<br>- 1.0 ml of **NaCl (250X stock; 21.9g/0.1L)**<br>- add **DMEM (cat#11965) to 250ml;** filter sterilize<br>- keep fresh for up to 2 weeks
**LTR medium -500ml**
- 125 ml **F12 (cat#11765)**<br>- 50ml's **FBS (qualified-grade)**.<br>- 10 ml's **B27** (regular)<br>- 5 ml's **Glutamax** (100X)<br>- 5 ml's **NEAA** (100X)<br>- 5 ml's **pyruvate** (100X)<br>- 500µl's **taurine** (1,000X - 1M stock)<br>- Add **DMEM (cat#11965)** to 500ml; filter sterilize.<br>- keep fresh for up to 2 weeks

(11) **Day 12** -Switch to **LTR medium** + 100nM SAG. Feed every other day until D18. When feeding, gently tilt the plate and aspirate the media by vacuum being careful not to suck up the vesicles. Add 10ml fresh medium directly to the plate.

(12) **Day 18** -Switch to **LTR medium (No SAG)**.

Days 20-160: Retinal differentiation/maturation.

(13) **Day 20** -Switch to **LTR medium** plus 500nM retinoic acid (ATRA). Optional - **Transfer to 40%O2/5%CO2.**

(14) **Day 29** -Add LTR medium plus 10μM DAPT until **D42**.
✪ recommended - Every few days groom the cultures by removing anything that doesn't look like a 3D OV; separate OV's if they stick together and periodically swirl plates to mix nutrients / metabolic waste. They should be spaced out at this point. Grooming is necessary for the first 2-3 months.
✪ note- its not uncommon for some excised vesicles to die during the first several months. The good 3D translucent cups should remain healthy so just keep removing the bad ones. Many structures also begin to form RPE. Those can remain.

(15) **Day 120** -ATRA is no longer added to the cultures.
✪ recommended - since outer segments will soon be forming feed the RCs very gently to avoid physical damage.

(16) **Day 150** -Outer-segment-like structures can often be observed by now.

Handling and Aliquoting of Reagents:

All-trans retinoic acid (ATRA) ✪- light sensitive
(20,000x- 10 mM): (Sigma #R2625-50MG)
10 mM stock: 5mg of ATRA to 1.66 ml DMSO. Store aliquots at -80C for up to 3-4 months. Working concentration = 500nM: Add 2 µl's per 40 ml's of LTR medium.

blebbistatin (2000x stock = 10mM; working concentration = 5µM).
- dissolve 1mg blebbistatin into 340µl DMSO, aliquot into 10µl aliquots (store at -80ºC). Add 10µl blebbistatin to every 20ml's mTeSR1.

DAPT (1,000x-10mM); working concentration: 10 µM.
- Add 10mg DAPT to 3.32 ml DMSO. Store aliquots at -20 to -80ºC away from light.

IWR-1-endo (Wnt Antagonist) (FW-409.4) **✪- light sensitive** - (EMD Millipore/Calbiochem #681669-10MG)
Working concentration - 3µM; 1,000X concentrated stock 3 mM in DMSO
- Add 10mg powder to 8.1ml DMSO. Store at -80C. * protect from light.

Matrigel (GF reduced) -each lot has a different concentration(normally between 8-10 mg/ml) so check lot# here: http://regdocs.bd.com/regdocs/searchCOA.do . Thaw 10ml bottle overnight on wet ice @ 4ºC, make 1MG aliquots, then store at -80ºC.
✪- Important- MG must be kept ice cold on wet ice at all times.

For coating: To coat plates: Add 10 ml's ice cold DMEM/F12 (can add 12 ml's for convenience) into a 15ml tube, pipet up and down 3-4x with a P1000 to cool tip, take up 1ml cold medium and transfer to Matrigel aliquot. Transfer contents back to the 15ml tube, mix and add 1ml per well of a 6-well plate. Coat overnight at 37ºC (8-16hrs is best).

For differentiation: Use final 1% (vol/vol) - add 100µl's MG into 10ml's BE6.2 medium (this is close to 0.1mg/ml). * On D1 add 200µl's MG so that when you add this to the initial 50µl's of cells it becomes 1% final. When adding IWR1e first prewarm BE6.2/Matrigel mixture for 10-15min.

Smoothened agonist SAG (1,000X stock - 100µM) (FW:599); Working concentration - 100nM.
- dissolve 1MG into 16.7ml DMSO, then make 100µl aliquots and store at -80C.

Sodium Chloride (NaCl; 250X stock):
- add 21.9g NaCl to 100ml cell culture grade water; filter sterilize after its added to the BE6.2 medium.

Taurine - working 1mM (400X stock - 400mM)(Sigma - T-8691; MW=125.15l)
- add 500mg of taurine into 10ml sterile cell culture grade distilled water; freeze these as 1.25ml aliquots at -20C. Use entire aliquot per 500ml bottle of LTR.

Catalog #	Description	Size	Source
Cell culture			
A6964-100ml	Accutase	100ml	Sigma
17504044	B27 supplement	10ml	Gibco
12587010	B27 supplement w/out Vitamin A	10ml	Gibco
11765	F12, with glutamine	500ml	LifeTech
11965-092 (1)<br>11965-118 (10)	DMEM (1X), liquid (+ 4.5g/L glucose, + L-glutamine, no pyruvate, no HEPES, + phenol red);	500ml<br>10x500ml	LifeTech
11360-070	100X pyruvate (100mM)	100ml	LifeTech
35050061	100X Glutamax	100ml	LifeTech
11140-050	100X MEM-NEAA	100ml	LifeTech
16140071	FBS - Heat inactivated (Qualified)	500ml	LifeTech
354230	Growth Factor Reduced BD Matrigel™ Matrix	10ml	BD Biosciences
Corning No.:3262	10cm well plates; ** for low attachment	20 pack	Corning
Lipidure®-Coat Plate A-U96	96 well Lipidure®-Coat Plate A-U96	7/pk	NOF Corporation
#05850	mTeSR1	500ml	Stem cell tech
25080094	Sodium bicarbonate		Mediatech
Small molecules			
A8960-5G	L-ascorbic acid-2-phos. mag.	5G	Sigma
R2625-50MG	ATRA, all-trans-Retinoic acid	50mg	Sigma
B0560-1MG	blebbistatin	1mg	Sigma
565770-5mg<br>565770-10mg	DAPT N-[N-(3,5-Difluorophenacetyl-L-alanyl)]-S-phenylglycine t-Butyl Ester- 'gamma-secretase inhib IX'	10mg	calbiochem
T0665-500MG	Holo-transferrin	500MG	Sigma
11376497001	Insulin	100 mg powder	Roche
681669	IWR-1-endo (Wnt Antagonist)	10mg	EMD Millipore
# 364590-63-6	SAG - Smoothened Agonist	1MG	EMD-Millipore
S5261-10G	Sodium Selenite	10 g powder	Sigma
T-8691 / T-0625	Taurine		Sigma

Supplemental Table 1. Information on primary antibodies (See Materials and Methods).

Antibody	Immunogen used	Source	Dilution IHC
Brn3 (C-13)	C-terminus of Brn-3b of human origin. (recognizes Brn3a, -b, -c)	sc-6026, goat polyclonal	1:200
LIM1+2	amino acid residues 1-360 of rat Lim2 protein	DSHB, mouse monoclonal, 4F2	1:20
Nanog	Synthetic peptide from human Nanog.	Millipore #AB9220;rabbit polyclonal	1:500
NF200	Neurofilament-200 from bovine spinal cord.	Sigma#N4142, rabbit polyclonal	1:200
OCT4	Synthetic peptide of human OCT4 within 300 residues of the C-terminus.	Abcam #ab19857; rabbit polyclonal	1:1,1000
Opsin rod RET-P1	Membrane preparation from adult rat retina.	Genetex# GTX23267, mouse monoclonal	1:500
Opsin R/G	Recombinant human red/green opsin.	Millipore#AB5405, rabbit polyclonal	1:1,000
Opsin S	Recombinant human blue opsin.	Millipore #AB5407, rabbit polyclonal	1:500-1,000
OTX2	Full length recombinant human OTX2 (excluding the first 5 amino acids).	Millipore #AB9566, rabbit polyclonal	1:500
Pax6	peptide QVPGSEPDMSQYWPRLQ from the C-terminus of mouse Pax6	Covance, rabbit polyclonal, PRB-278P	1:2,000
Pax6	amino acids 1–223 of chicken Pax6	DSHB, mouse monoclonal, Pax6-concentrate	1:100
PNAL	Peanut agglutinin Lectin	Invitrogen	1:1,000
PSD95 (clone K28/43)	amino acids 77-299 of the human PSD95.	UC Davis NeuroMab Facility #75-028, mouse monoclonal,	1:1,000 - 1,1500
Recoverin	Recombinant human recoverin	Millipore #AB5585, rabbit polyclonal	1:2,000
Ribeye (CtBP2/ribeye)	amino acids 361-445 from the C-terminal region of mouse CtBP2.	BD-Biosciences , mouse monoclonal, 612044	1,000
SOX2	Synthetic peptide within 300 residues of the C-terminus of human SOX2.	SOX2 (Abcam #ab97959, rabbit polyclonal.	1:1,000
SSEA4; (clone MC-813-70)	Human embryonal carcinoma cell line 2102Ep	Millipore #MAB4304; mouse monoclonal	1:250

Supplemental Table 2. Oligonucleotides for qPCR detection of human retinal genes.

Gene name	Gene ID	Name	Sequence	amplicon size (bp)
B-actin	GeneID: 60	beta-actin(103)F	GCGAGAAGATGACCCAGATC	103
		beta-actin(103)rev	CCAGTGGTACGGCCAGAGG	
CREBBP	GeneID: 1387	CREBBP_F	GAGAGCAAGCAAACGGAGAG	189
		CREBBP_rev	AAGGGAGGCAAACAGGACA	
FBXL12	GeneID: 54850	FBXL12_F	GCCTTGGTCATATCATCAG	176
		FBXL12_rev	TTCTTCATCCGTCCTGTT	
SRP72	GeneID: 6731	SRP72_F	TCTGCCTCTACAAGTAACATCAT	118
		SRP72_rev	CTCATCACCAGCCACCTT	
Arrestin	GeneID: 407	hArrestin(94)F	AGGAAAGCCCTGTGGGATTGACTT	94
		hArrestin(94)rev	AACCAGCCGCACATAGTCTCTCTT	
CNGA3	GeneID:1261	hCNGA3_112_F	AACTTTGGCAGGCAATCATCTGGG	112
		hCNGA3_112_rev	TGAGGTCTTTACCTTGAGGTGGGT	
CNGB1	GeneID: 1258	hCNGB1_105_F	TCGCCATCGACGTGAACTACAACA	105
		hCNGB1_105_rev	AGACAACAGAGCGAAGCCTCTTCA	
CNGB3	GeneID: 54714	hCNGB3_106_F	AGTGCCAGAGCAGAAGGAAATGGA	106
		hCNGB3_106_rev	TAGCTGGGCATCGGCATACTCATT	
CRX	GeneID: 1406	hCRX_142_F	TGTTTGCCAAGACCCAGTAC	142
		hCRX_142_rev	TGCTGTTTCTGCTGCTGTCGC	
NR2E3	GeneID: 10002	hNr2E3-151_F	GCCTGGACAGCATCCATGAG	151
		hNr2E3-151_rev	ATGGCCCCGAGGAGAAAGAG	
NRL	GeneID: 4901	hNRL_147_F	AGGCTCGCTGTGACCGGCTA	147
		hNRL_147_rev	TGCAGAGAACCGTGCAGCCG	
Opsin-Rhodopsin	GeneID: 6010	hqRhod188_F	TCATGGTCCTAGGTGGCTTC	188
		hqRhod188_rev	GGAAGTTGCTCATGGGCTTA	
Opsin-Short **λ**	GeneID: 611	hOPN1SW127_F	CGGCTTGTCACCATTCCTTC	127
		hOPN1SW127_rev	CTGTCATGGCCTTCCCACAC	
Opsin-Medium **λ**	GeneID: 728458	hMW_unique_F	TCACCCCACTCAGCATCATCGTGCT	152
		hMW_unique_rev	GAAGCAGAATGCCAGGACCATCAC	
Opsin-Long **λ**	GeneID: 5956	hLW_unique_F	CATCATCCCACTCGCTATCATCATGC	154
OTX2	GeneID: 5015	hOTX2_190_F	AGAGCAGCCCTCACTCGCCA	190
		hOTX2_190_rev	AGTCGGCCCAAATCGGGGGT	
Pax6	GeneID: 5080	hPax6+5a(130)F	CTCGGTGGTGTCTTTGTCAAC	130
		hPax6+5a(130)rev	ACTTTTGCATCTGCATGGGTC	
Rax	GeneID: 30062	hRax(81)F	AGCGAAACTGTCAGAGGAGGAACA	81
		hRax(81)rev	TCATGCAGCTGGTACGTGGTGAAA	
Recoverin	GeneID: 5957	hRCVRN(179)F	ACCGCGGGCAAGACCAACCA	179
		hRCVRN(179)rev	TCGGCTCGCTTTTCCGGCGTG	
SIX6	GeneID: 4990	hqSIX6(198)F	ACCCCTACGCAGGTGGGCAA	198
		hqSIX6(198)rev	TGAAGTGGCCGCCTTGCTGG	
VSX2	GeneID: 338917	hVSX2(122)F	GGCGACACAGGACAATCTTTA	122
		hVSX2(122)rev	TTCCGGCAGCTCCGTTTTC	
